# Systemic reprogramming of monocytes in Crohn’s disease promotes their intestinal inflammatory function

**DOI:** 10.1172/jci.insight.192830

**Published:** 2026-08-11

**Authors:** Eve Hornsby, Radha Gadhok, Inva Hoti, Eva Wozniak, James R. Boot, Emma Connick, Paul Stevens, Holly Creed, Amy Lewis, Andrew Silver, James O. Lindsay, Andrew J. Stagg

**Affiliations:** 1 Centre for Immunobiology and Infection, Blizard Institute, Barts and The London School of Medicine and Dentistry, Queen Mary University of London, London, United Kingdom.; 2 Department of Gastroenterology, Barts Health NHS Trust, The Royal London Hospital, London, United Kingdom.; 3 Genome Centre and; 4 Centre for Genomics and Child Health, Blizard Institute, Barts and The London School of Medicine and Dentistry, Queen Mary University of London, London, United Kingdom.

**Keywords:** Immunology, Inflammation, Monocytes

## Abstract

Bone marrow–derived circulating monocytes continuously replenish intestinal macrophages, which become dysregulated in inflammatory bowel disease (IBD) and contribute to disease pathology. The origins of this dysregulation remain poorly understood. Here, we investigate the reprogramming of circulating monocytes in IBD prior to tissue recruitment using single-cell transcriptomic, epigenomic, and functional approaches. We characterize blood monocyte heterogeneity in newly diagnosed, treatment-naive IBD patients and healthy controls and show that monocytes in Crohn’s disease (CD) display a distinct transcriptional profile and altered distributions across inferred developmental trajectories; less pronounced changes were observed in ulcerative colitis (UC). We link CD-associated transcriptional changes to alterations in chromatin accessibility and identify NF-κB, EGR, KLF, and AP-1 family transcription factors as putative regulators of an inflammatory gene program in blood monocytes from patients with CD. We uncover a potential role for IFN-γ in priming blood monocytes for inflammatory function in CD by limiting their capacity to be regulated by IL-10. Finally, we show that the transcriptional and functional alterations in monocytes from patients with CD are maintained in monocyte-derived cells from the intestine. Together these data suggest that intestinal macrophage dysfunction in CD is, at least in part, pre-established by systemic signals prior to tissue recruitment.

## Abstract 2

Intestinal macrophage dysfunction in Crohn’s disease is partly pre-established in circulating monocyte precursors before tissue recruitment.

## Graphical abstract

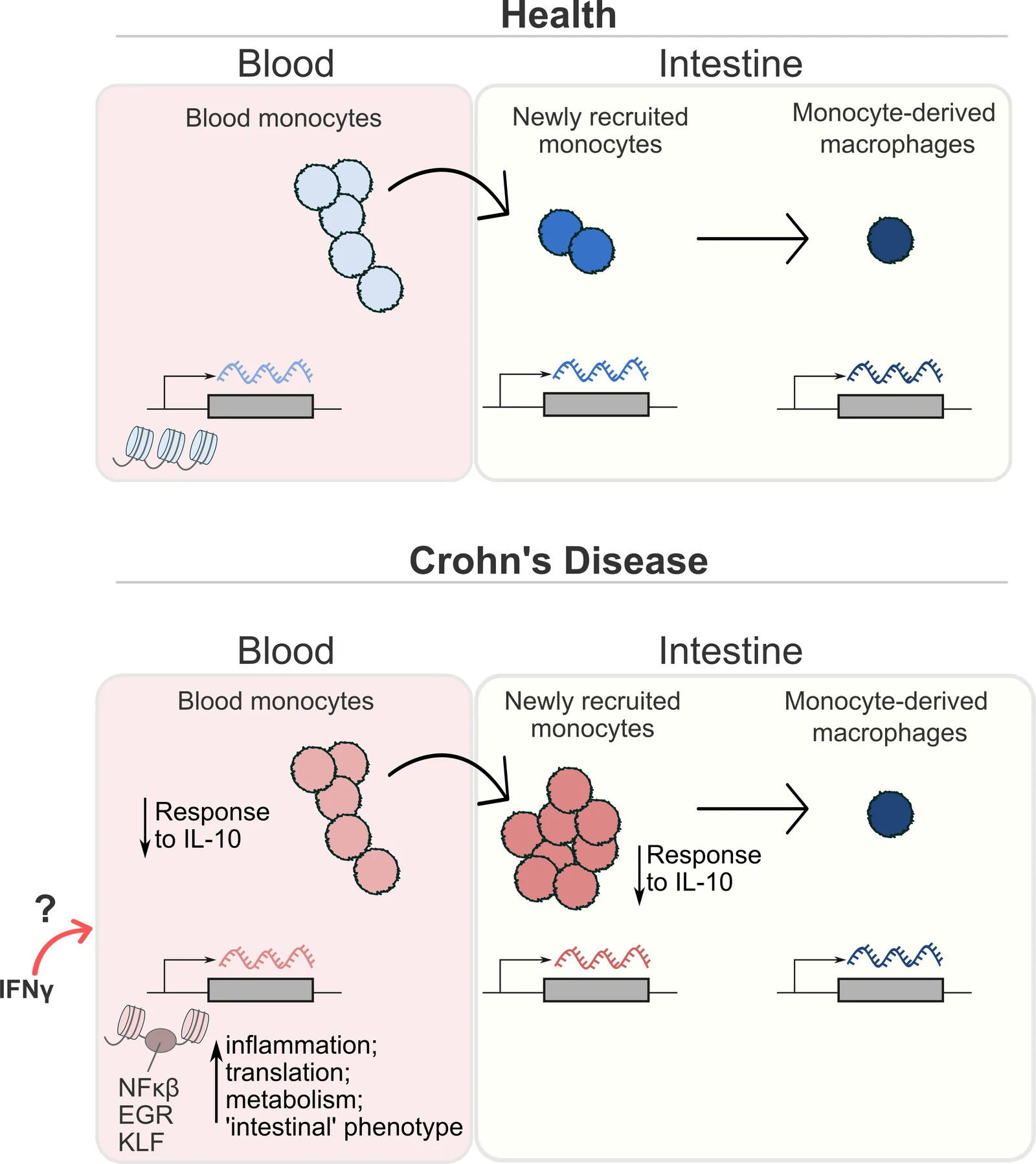

## Introduction

Inflammatory bowel disease (IBD) is a chronic inflammatory disease of the gastrointestinal tract that includes Crohn’s disease (CD) and ulcerative colitis (UC). Although numerous therapeutic options are available, a substantial proportion of patients fail to achieve durable clinical remission (1). This highlights a critical unmet need to elucidate the mechanisms driving disease pathogenesis and to develop more effective, targeted therapies.

Intestinal macrophages possess antiinflammatory, bactericidal, phagocytic, and autophagic properties that regulate the immune response to commensal microbiota and are essential for the maintenance of healthy tissue homeostasis. In adulthood, the majority of these macrophages are replenished continuously by bone marrow–derived blood monocytes, which differentiate into mature macrophages within the tissue (2, 3). Loci associated with susceptibility to IBD are strongly enriched for genes involved in monocyte/macrophage differentiation and function (4), and monocyte-derived cells that produce pathological cytokines such as TNF-α, IL-1β, and IL-23 accumulate in the inflamed intestine of patients with IBD (5–8).

Signals from the tissue microenvironment influence the differentiation and function of newly recruited monocytes. In health, they mature into regulatory macrophages under the influence of local signals including TGF-β and IL-10 (9, 10), while monocytes infiltrating the inflamed intestine are redirected to adopt inflammatory properties (11, 12). Mice lacking the IL-10 receptor (IL-10R) specifically in intestinal macrophages develop spontaneous colitis (10, 13), suggesting that the ability of macrophages to respond to IL-10 is a nonredundant mechanism limiting inflammatory responses to the commensal microbiota. Consistent with this concept, children with loss-of-function mutations in genes encoding IL-10R develop an aggressive form of IBD early in life (14).

Emerging evidence suggests that before tissue recruitment, systemic signals can shape monocyte fate and function. Cytokines, alarmins, and microbial products can initiate emergency myelopoiesis, leading to enhanced output of inflammatory monocytes from bone marrow and extramedullary sites (15). Furthermore, epigenetic reprogramming of monocyte progenitors upon infection results in long-term functional changes to their derivatives and an altered response toward secondary infection in a phenomenon termed “trained immunity” (16). In mouse studies, dysregulated monocyte progenitor activity promotes colitis in an IFN-γ– and GM-CSF–dependent manner (17), and IFN-γ educates monocyte progenitors for enhanced function in acute gastrointestinal infection (18). The extent of systemic monocyte reprogramming in IBD and its influence on intestinal macrophage function, however, are currently unknown.

Circulating monocytes are broadly divided into three subsets based on expression of CD14 and CD16, although multidimensional single-cell techniques are beginning to identify further heterogeneity (19). Classical monocytes (CD14^+^CD16^–^) exit the bone marrow and circulate briefly before either differentiating into blood-resident nonclassical (CD14^lo^CD16^+^) monocytes via an intermediate (CD14^+^CD16^+^) stage, or migrating into tissues (20). Ly6C^hi^CCR2^+^ monocytes, which are the murine equivalent of human classical monocytes, give rise to macrophage subsets in both healthy and inflamed colon (2), and recently recruited blood monocytes within IBD mucosa resemble classical monocytes (6, 21). Intermediate monocytes are expanded in patients with CD (6); however, a detailed analysis of blood monocyte heterogeneity in IBD has not been documented.

Here, we use single-cell RNA-seq (scRNA-seq) to define blood monocyte heterogeneity and potential developmental pathways in a cohort of newly diagnosed, treatment-naive CD and UC patients compared with healthy controls (HCs). We establish the scope of IBD-associated transcriptional alterations in blood monocyte populations and demonstrate preservation of these changes in intestinal monocyte-derived cells from inflamed tissue. We also use single-nucleus multiomics to link CD-associated gene expression profiles in blood monocytes with altered chromatin accessibility and therefore predict putative transcription factor regulators of this gene expression. Finally, we show that in CD, monocytes are primed for inflammatory function before arrival in the intestinal mucosa, as reflected by impaired IL-10–mediated suppression of TNF-α.

## Results

### Circulating monocytes exhibit heterogeneity in health and IBD.

**Figure 1:**
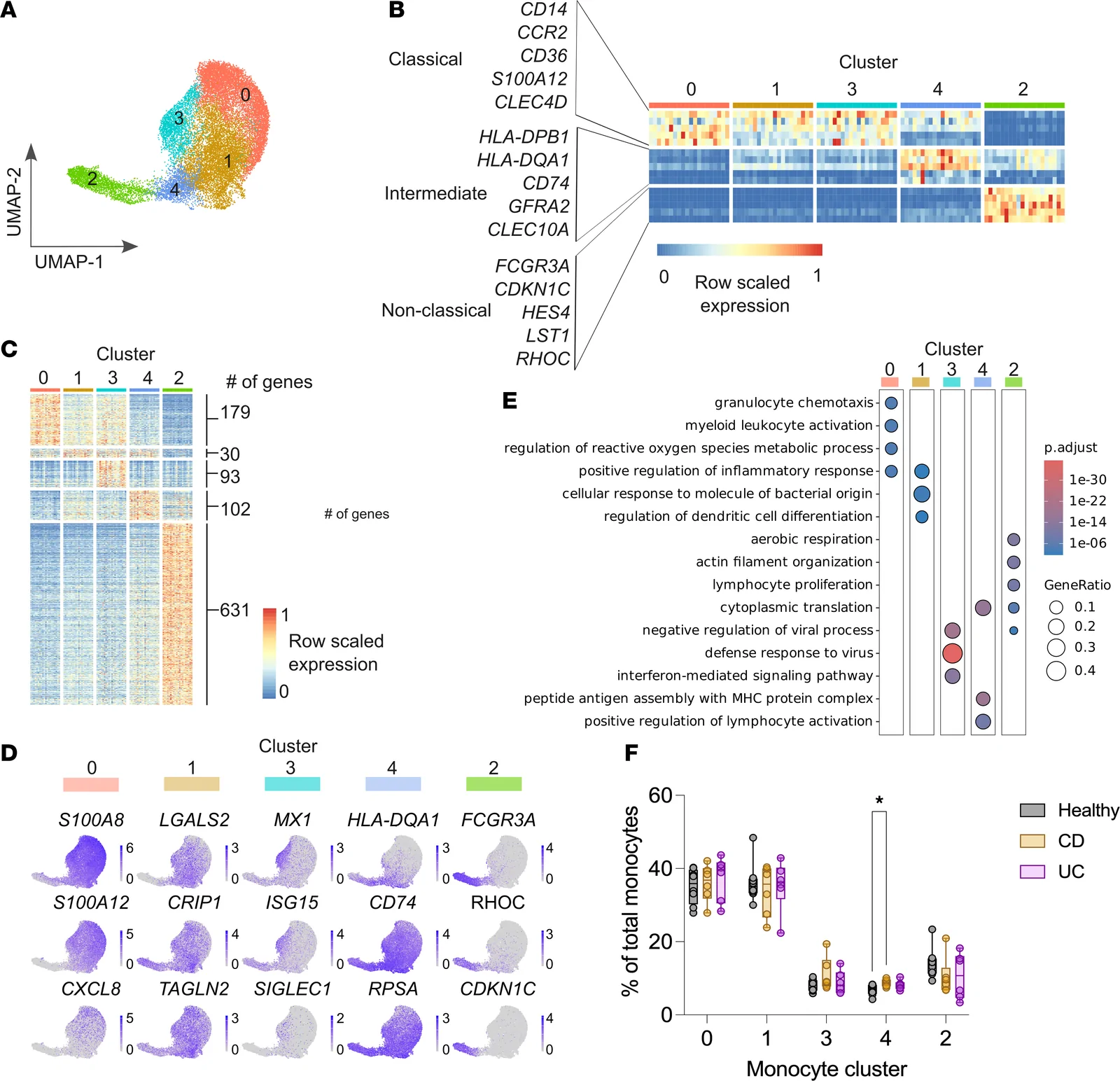
Circulating monocytes exhibit heterogeneity in health and IBD. scRNA-seq analysis of peripheral blood monocytes from newly diagnosed, treatment-naive IBD patients with active disease (CD, *n* = 6; UC, *n* = 6) and HCs (*n* = 8). (**A**) UMAP projection of monocyte clusters from all integrated donors. (**B**) Average cell expression per donor per cluster of distinguishing markers of classical, intermediate, and nonclassical monocytes. (**C**) Average cell expression per donor per cluster of cluster-defining markers. (**D**) UMAP projections showing log-normalized expression of select cluster-defining markers. (**E**) Top 3 significantly enriched Gene Ontology (biological process) terms for cluster-defining markers after removal of redundant terms. Gene ratio, representing the number of genes in the term divided by the total number of defining genes for that cluster, and adjusted *P* value are indicated. (**F**) Proportions of each cluster expressed as a percentage of total monocytes per donor. *P* value denotes the result of 2-way ANOVA with Dunnett’s correction. Box-and-whisker plots show the median (center line), 25th and 75th percentiles (box), and minimum and maximum values (whiskers). Individual data points are overlaid. *P < 0.05.

To identify IBD-associated changes in circulating monocytes, we performed scRNA-seq on monocytes enriched from the peripheral blood of 12 patients with newly diagnosed IBD (CD, *n* = 6; UC, *n* = 6) and 8 age-, ethnicity-, and sex-matched HCs. Patients with IBD had clinical and biomarker evidence of active disease and were all treatment naive. The CD cohort comprised 3 patients with colonic, 2 with ileal, and 1 with ileocolonic disease. Four patients had nonstricturing, nonpenetrating disease, 1 had stricturing disease, and 1 had penetrating disease; 2 patients also had perianal involvement. The UC cohort included 3 patients with left-sided colitis and 3 with extensive colitis (Supplemental Data File 1; supplemental material available online with this article; https://doi.org/10.1172/jci.insight.192830DS1). Monocytes for the scRNA-seq analysis were enriched using negative magnetic selection. After sample integration and exclusion of minor clusters expressing known markers of lymphocytes, dendritic cells, platelets, and hematopoietic stem cells (HSCs), graph-based clustering identified 5 transcriptionally distinct monocyte clusters within a single object of 19,967 cells derived from both patients with IBD and HCs (Figure 1A and Supplemental Figure 1A). Expression of previously identified distinguishing markers of circulating monocyte subsets (22, 23) indicated that clusters 0, 1, and 3 mapped to classical monocytes while clusters 2 and 4 represented nonclassical and intermediate monocytes, respectively (Figure 1B). A more extensive analysis of cluster-discriminating genes (Figure 1, C–E, and Supplemental Data File 2) revealed that cluster 0 (classical) was defined by an inflammatory and migratory signature and characterized by expression of genes such as *S100A8/12* and *CXCL8*. Cluster 1 (classical) had increased expression of a small subset of genes with previously described proinflammatory roles in monocytes, including *LGALS2*, *CRIP1*, and *TAGLN2* (24–26), while cluster 3 (classical) was uniquely defined by interferon signaling–related genes such as *MX1*, *ISG15*, and *SIGLEC1*. Consistent with well-established characteristics of intermediate and nonclassical monocytes (22, 23), cluster 4 (intermediate) had elevated expression of genes involved in antigen presentation (e.g., *HLA-DQA1*, *CD74*) and translation (e.g., *RPSA*), while cluster 2 (nonclassical) was defined by expression of genes such as *FCGR3A*, *CDKN1C*, *MS4A1*, *TCF7L2*, *IFITM3*, and *IFITM2*, in addition to genes enriched for actin-related pathways (e.g., *ACTB*, *RHOC*, and *CDC42*). The 5 monocyte clusters were present at similar frequencies in both patients with IBD and HCs, although there was a small but significant increase in cluster 4 (intermediate) monocytes in CD relative to HCs (Figure 1F) in line with the previously reported expansion of intermediate monocytes in CD (6).

### Predicted monocyte differentiation trajectories are altered in CD.

**Figure 2:**
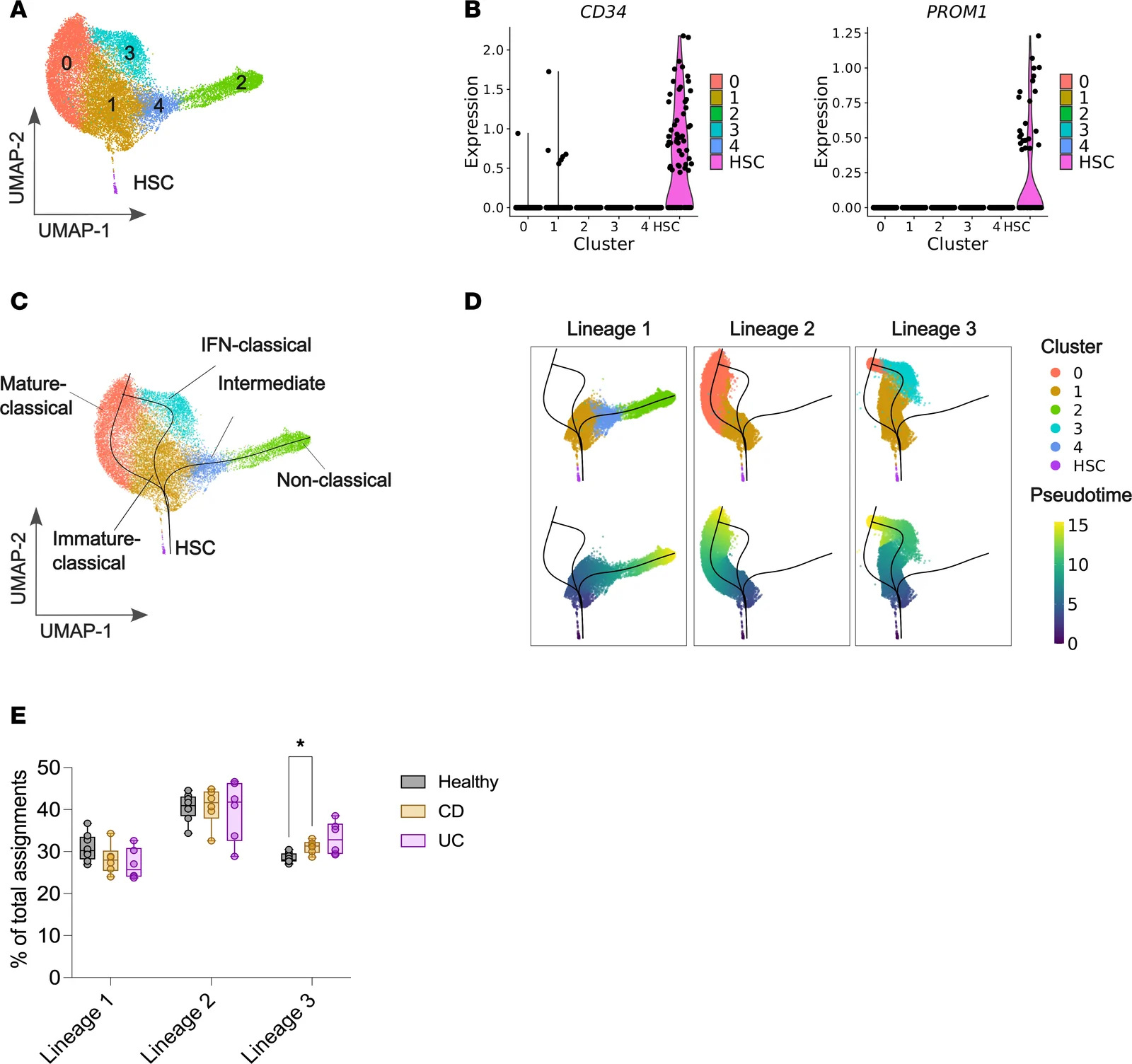
Predicted monocyte differentiation trajectories are altered in CD. (**A**) UMAP projection of monocytes (clusters 0–4) and hematopoietic stem cells (HSCs) from all integrated donors. (**B**) Log-normalized expression of HSC markers. (**C**) Global lineages of monocyte differentiation as determined by Slingshot, using HSCs as a starting cluster. (**D**) Individual global lineages of monocyte differentiation; clusters and pseudotime values for each lineage are indicated. (**E**) Proportion of cells assigned to each lineage expressed as a percentage of the total assignments per donor. *P* value denotes the result of 2-way ANOVA with Dunnett’s correction. Box-and-whisker plots show the median (center line), 25th and 75th percentiles (box), and minimum and maximum values (whiskers). Individual data points are overlaid. *P < 0.05.

Given that circulating monocyte subsets have been linked by a linear developmental trajectory (20), we explored how the 5 monocyte clusters are developmentally related and how this relationship is altered in IBD. To this end, we performed trajectory analysis on the scRNA-seq dataset using Slingshot (27). We re-incorporated a cluster of *CD34^+^PROM1^+^* HSCs (28) that were filtered out in our initial analysis to be used as a starting cluster. Reclustering of the new dataset identified 5 clusters of monocytes with almost identical features to those described in the initial analysis plus an additional sixth cluster representing the progenitors (Figure 2, A and B, and Supplemental Figure 1B). Pseudotime ordering (Figure 2, C and D) positioned cluster 1 (classical) monocytes immediately after the progenitors, suggesting they are the most immature monocyte cluster. This is consistent with their lower expression of canonical “classical monocyte” genes (Figure 1B), such as *S100A12*, *CCR2*, and *CD36*, relative to other classical monocytes. From this cluster, cells were mapped along 3 predicted lineage trajectories (Figure 2, C and D). In lineage 1, cells transitioned to cluster 2 (nonclassical) via cluster 4 (intermediate) consistent with the established blood monocyte differentiation pathway. In lineages 2 and 3, cluster 1 (classical) monocytes gave rise to putatively more mature classical monocytes (cluster 0), with lineage 3 passing through an interferon response gene–expressing state (cluster 3). There were no gross alterations in the distribution of cells across each of these predicted differentiation trajectories in health versus IBD (Supplemental Figure 1C); however, there was a small increase in the proportion of cells assigned to lineage 3 in CD compared with HCs, and a concomitant nonsignificant reduction in lineage 1 (Figure 2E). This effect was also observed in UC, although it did not reach statistical significance. These data are suggestive of a subtle shift in the representation of monocyte lineages in CD, consistent with an increased contribution from an inferred trajectory leading to mature classical monocytes, which are considered the precursors to intestinal macrophages (2, 6, 7, 21).

### Circulating monocytes from patients with CD display a distinct transcriptional profile.

**Figure 3:**
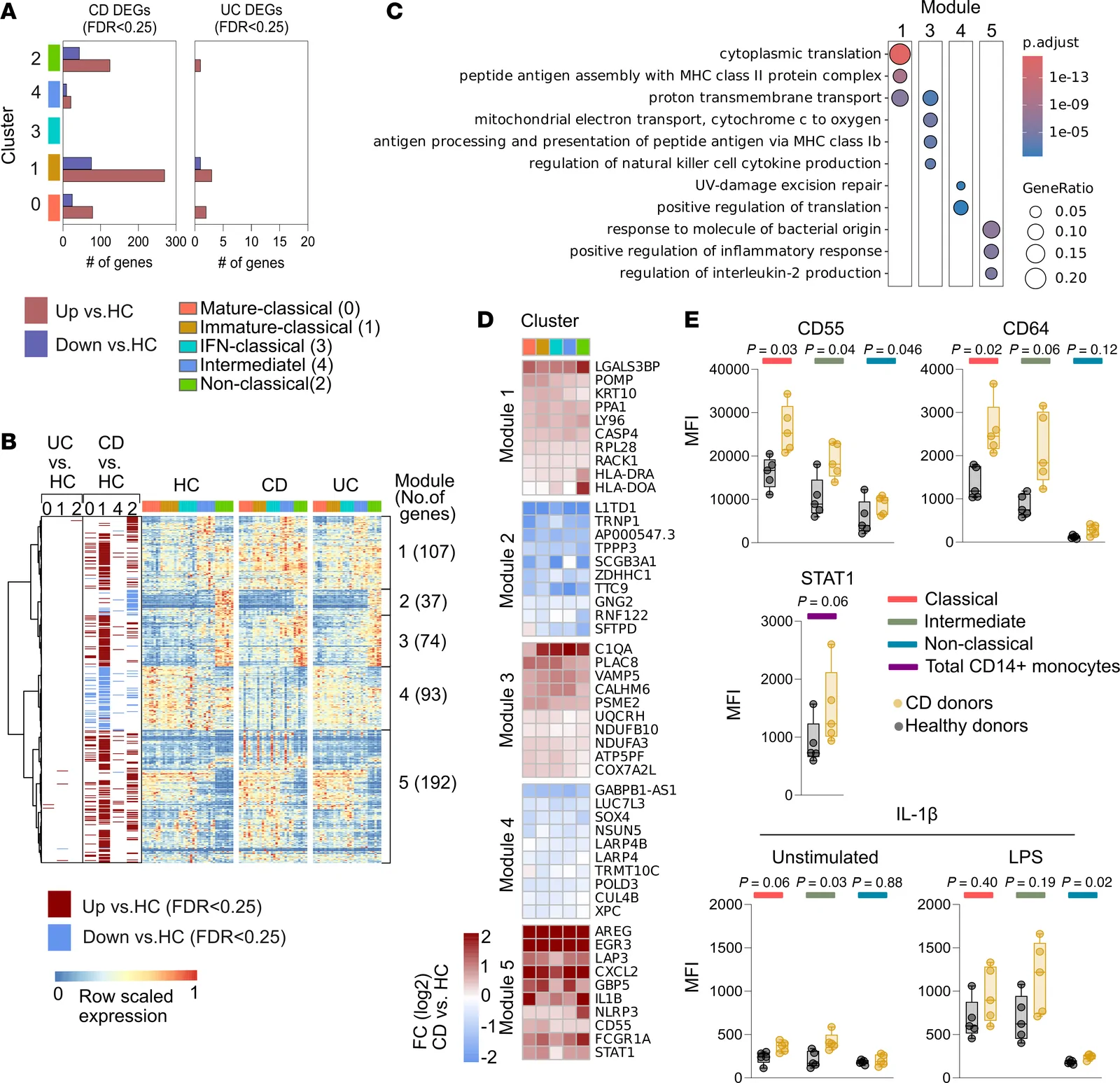
Circulating monocytes from patients with CD display a distinct transcriptional profile. Pseudobulk differential expression analysis was performed on monocyte clusters from treatment-naive patients with CD (*n* = 6) and UC (*n* = 6) versus HCs (*n* = 8). (**A**) Numbers of genes with FDR ≤ 0.25 are plotted for each cluster and disease. (**B**) Hierarchical clustering of differentially expressed genes (DEGs) based on average cell expression per donor per cluster. Cluster and disease type with FDR ≤ 0.25 and the direction of regulation are shown in the left annotation. (**C**) Top 3 significantly enriched Gene Ontology (biological process) terms for each module of DEGs after removal of redundant terms. Gene ratio (genes in term/total genes in module) and adjusted *P* value are indicated. (**D**) Ten pathway genes for each module of DEGs, expressed as log_2_ FC in CD compared with HC within each cluster. (**E**) Flow cytometry analysis of monocytes in a separate cohort of treatment-naive patients with CD (*n* = 5) and HCs (*n* = 5). Top: Mean fluorescence intensity (MFI) of extracellular CD55 and CD64 in classical, intermediate, and nonclassical monocytes. Middle: MFI of intranuclear STAT1 in total CD14^+^ monocytes. Bottom: MFI of intracellular IL-1β in classical, intermediate, and nonclassical monocytes following a 3-hour stimulation of PBMCs with 0.4 ng/mL lipopolysaccharide (LPS) or an unstimulated control. MFIs are normalized within each sample by subtraction of the MFI of the relevant isotype control (for CD55, CD64, and STAT1) or of the HLA-DR^–^ population (for IL-1β). *P* values were determined by 2-way ANOVA with Šidák’s correction (for CD55, CD64, and IL-1β) or Mann-Whitney test for STAT1. Box-and-whisker plots show the median (center line), 25th and 75th percentiles (box), and minimum and maximum values (whiskers). Data points are overlaid.

To identify gene expression changes in blood monocytes from patients with IBD compared with HCs, we performed pseudobulk differential expression analysis in each of the 5 monocyte clusters. Pseudobulk approaches provide higher precision and specificity than models that treat individual cells as independent observations; however, they also have reduced sensitivity (29). To account for this trade-off, we applied a relaxed false discovery rate (FDR) cutoff of 0.25. Despite this, monocytes from patients with UC showed few differentially expressed genes (DEGs) compared with HCs, whereas CD monocytes exhibited widespread transcriptional changes, most prominently in the putative immature classical monocyte cluster (cluster 1). There were no DEGs in the IFN-classical cluster (cluster 3) (Figure 3A and Supplemental Data File 2). Many DEGs were shared across monocyte subsets, with consistent fold changes (FCs) observed even where statistical significance was not reached (Supplemental Figure 1, D and E). UC monocytes showed similar directional changes to many CD-associated DEGs, but with reduced magnitude and limited statistical significance (Supplemental Figure 1, D and E). Notably, the magnitude of CD-associated transcriptional changes in monocyte clusters correlated with serum C-reactive protein (CRP) in CD but not UC, indicating that in CD this monocyte signature is linked with systemic inflammation (Supplemental Figure 2).

Hierarchical clustering of all DEGs identified distinct modules of genes that were broadly organized by differential regulation in CD and by variation in expression across monocyte subsets (Figure 3B). Genes upregulated in CD were predominantly found in modules 1, 3, and 5, with the greatest impact observed in the immature classical monocyte cluster (cluster 1), as indicated by the high number of significant genes (Figure 3B). Functional enrichment analysis revealed that module 5 was associated with immune-related processes, including “response to molecule of bacterial origin” and “positive regulation of inflammatory response” (e.g., *LAP3*, *FCGR1A*, *GBP5*, *NLRP3*, *CD55*, *CXCL2*, *IL1B*). Module 1 was enriched for genes involved in “cytoplasmic translation” (e.g., *RPL28*, *RACK1*), while module 3 was associated with “proton transmembrane transport” (e.g., *UQCR10*, *NDUFB10*, *NDUFA3*, *COX7A2L*) (Figure 3, C and D, and Supplemental Data File 2).

In contrast, genes downregulated in CD monocytes were present mainly in modules 2 and 4. Module 2 did not show significant enrichment for specific Gene Ontology pathways but included genes such as the RNA-binding protein *L1TD1* and the DNA-binding protein *TRNP1*. This module was most affected in nonclassical monocytes, where expression of these genes was elevated relative to other monocyte clusters. Module 4 genes were most affected in the immature classical monocyte cluster (cluster 1) and were enriched for processes including “positive regulation of translation” (e.g. *NSUN5*, *LARP4B*) and “UV-damage excision repair” (e.g., *POLD3*, *CUL4B*) (Figure 3, C and D, and Supplemental Data File 2).

Gene set enrichment analysis (GSEA) (Supplemental Figure 3A), which does not depend on the selection of DEGs, corroborated these findings, demonstrating enrichment of inflammatory, cytokine production, protein processing, DNA repair, and metabolic pathways in CD monocytes, with a less pronounced effect in UC.

To validate key transcriptional findings at the protein level, we performed flow cytometry in an independent cohort of 5 treatment-naive CD patients with active disease and 5 HCs (Figure 3E and Supplemental Figure 3, B and C). Consistent with the transcriptomic data, CD monocytes exhibited increased surface expression of CD55 and CD64, most prominently within classical and intermediate subsets. A trend toward increased intranuclear STAT1 expression in total CD14^+^ monocytes was also observed. Intracellular IL-1β production was assessed following a 3-hour stimulation with lipopolysaccharide (LPS) or medium alone (unstimulated) and was modestly increased in CD monocytes compared with controls under both conditions. This increase reached statistical significance in unstimulated intermediate monocytes and LPS-stimulated nonclassical monocytes. In contrast, expression of HLA-DR, CD63, and CD87 was not different between health and CD (Supplemental Figure 3D). These results confirm that components of the inflammatory gene signature in circulating CD monocytes are partially reflected at the protein level.

Taken together these data demonstrate that blood monocytes in CD exhibit broad transcriptional reprogramming, whereas those from patients with UC are less affected.

### Changes in chromatin accessibility underpin the upregulation of a subset of inflammatory genes in CD blood monocytes.

**Figure 4:**
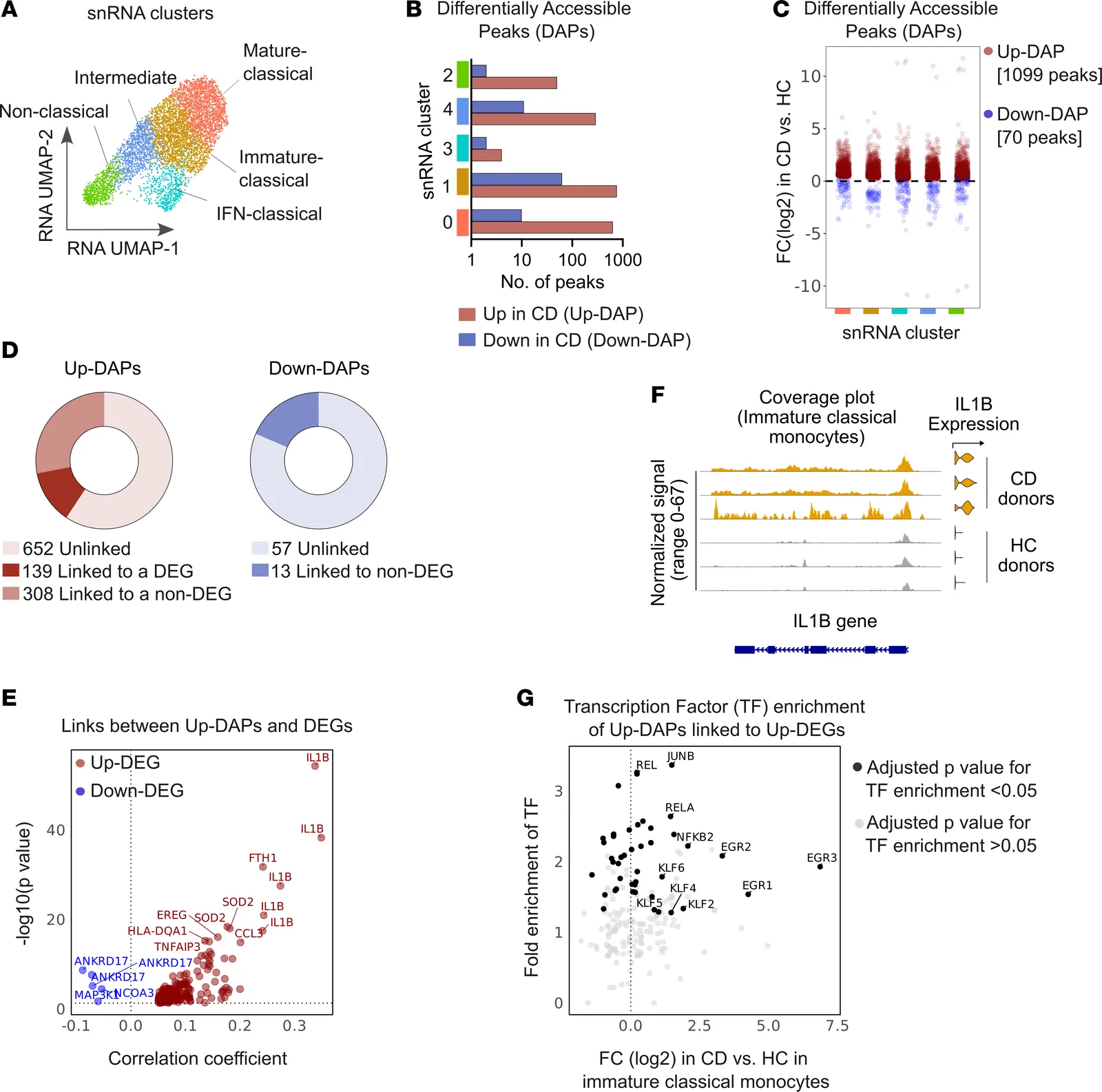
Changes in chromatin accessibility underpin the upregulation of a subset of inflammatory genes in CD peripheral blood monocytes. Single-nucleus multiomics (snRNA-seq and snATAC-seq) analysis of peripheral blood CD14^+^ monocytes from newly diagnosed, treatment-naive patients with active CD (*n* = 3) and HCs (*n* = 3). (**A**) UMAP projection of snRNA-seq monocyte clusters from all integrated donors. (**B**) Number of DAPs in each snRNA-seq cluster between health and CD. (**C**) log_2_ FC accessibility of DAPs (identified in at least 1 snRNA-seq cluster) plotted in all snRNA-seq clusters. (**D**) Numbers of DAPs that are either unlinked to gene expression, linked to a DEG, or linked to a non-DEG are shown. (**E**) Correlation coefficients and –log_10_ *P* values are plotted for the links between Up-DAPs and DEGs. (**F**) Coverage plot displaying normalized read density around the *IL1B* gene in immature classical monocytes in CD and HC donors. (**G**) Transcription factor (TF) motif enrichment analysis of Up-DAPs that are significantly linked to Up-DEGs. Fold enrichment is displayed against log_2_ FC expression in CD versus health for immature classical monocytes. Human TFs with log_10_ expression greater than 1 in any RNA cluster are shown, and significantly enriched motifs are indicated.

To identify regulatory elements underlying the altered transcriptional profile in monocytes from patients with CD, we examined a separate single-nucleus multiome dataset generated from CD14^+^ monocytes isolated from the peripheral blood of 3 newly diagnosed, treatment-naive patients with CD and 3 HCs (Supplemental Data File 1). The dataset, comprising RNA-seq and assay for transposase-accessible chromatin using sequencing (ATAC-seq), allowed us to profile gene expression and chromatin accessibility within the same cell. After the exclusion of minor clusters expressing known markers of lymphocytes and dendritic cells, we obtained a single dataset containing 5,892 single nuclei derived from both patients with CD and HCs that met quality control and filtering criteria. Graph-based clustering of the dataset revealed 5 single-nucleus RNA-seq (snRNA-seq) clusters of monocytes with resemblance to the clusters in the original scRNA-seq dataset as well as 4 single-nucleus ATAC-seq (snATAC-seq) clusters (Figure 4A and Supplemental Figure 4, A and B). While *FCGR3A^+^* nonclassical monocytes were distinguished in both clustering modalities, snRNA-seq clusters representing classical and intermediate monocytes did not overlap with snATAC-seq clusters, suggesting that there is limited association between the transcriptional and chromatin accessibility landscapes in these populations (Supplemental Figure 4A).

Differential analysis of chromatin accessibility between health and CD identified a total of 1,169 differentially accessible peaks (DAPs) that were significantly different (adjusted *P* < 0.05) in at least one monocyte cluster (Figure 4, B and C, and Supplemental Figure 4C). These 1,169 DAPs had a consistent direction of change across all monocyte clusters, even when statistical significance was achieved in only some, indicating a shared rather than subset-specific remodeling of chromatin in circulating monocytes. Ten thousand ninety-nine DAPs had increased accessibility in CD (Up-DAPs), while 70 had reduced accessibility (Down-DAPs).

To connect these DAPs to putative target genes, we next explored peak-gene associations across the full monocyte dataset. We defined DEGs as those that were CD DEGs in our original scRNA-seq analysis and that also displayed equivalent log_2_ FCs between health and CD in all snRNA-seq clusters (Supplemental Figure 5A). A large proportion of DAPs were not significantly associated with gene expression (Figure 4D), but Up-DAPs were in close proximity to genes involved in inflammatory and cytokine responses, indicating that monocytes from patients with CD have a poised chromatin state that may subsequently bias expression of genes involved in these pathways (Supplemental Figure 5, B and C). One hundred thirty-nine Up-DAPs were significantly correlated with DEG expression (Figure 4D), predominantly showing positive associations with a subset of Up-DEGs (Figure 4E) involved in antigen presentation, cell activation, and cytokine production, including *IL1B*, *SOD2*, and *CCL3* (Figure 4F and Supplemental Figure 6A). Five negative correlations were observed with Down-DEGs (*ANKRD17*, *NCOA3*, *MAP3K1*). Notably, some genes, including *IL1B*, *SOD2*, and *ANKRD17*, were linked to multiple Up-DAPs and therefore appear more than once (Figure 4E). Transcription factor motif enrichment analysis of Up-DAPs that were linked to Up-DEGs revealed members of the EGR family (EGR1, EGR2, EGR3), NF-κB family (NFKB2, REL, RELA), KLF family (KLF2, KLF6), and AP-1 family (JUNB), among others, as putative regulators of this subset of Up-DEGs, also based on their elevated expression in CD immature classical monocytes compared with HCs (Figure 4G). Similar expression patterns were also seen for the other clusters (data not shown). Notably, a proportion of DEGs were also unlinked to chromatin accessibility, suggesting that altered gene expression in CD monocytes is to some degree regulated independently of alterations to chromatin (Supplemental Figure 6B).

Together these data suggest that cross-subset changes in chromatin accessibility underpin the upregulation of a subset of inflammatory genes in blood monocytes from patients with CD. Furthermore, blood monocytes in CD have an altered chromatin state, rendering them poised for differential expression of genes involved in inflammation and cytokine signaling.

### CD monocytes are primed for inflammatory function before they arrive in the intestine, with evidence of IFN-γ–associated conditioning.

**Figure 5:**
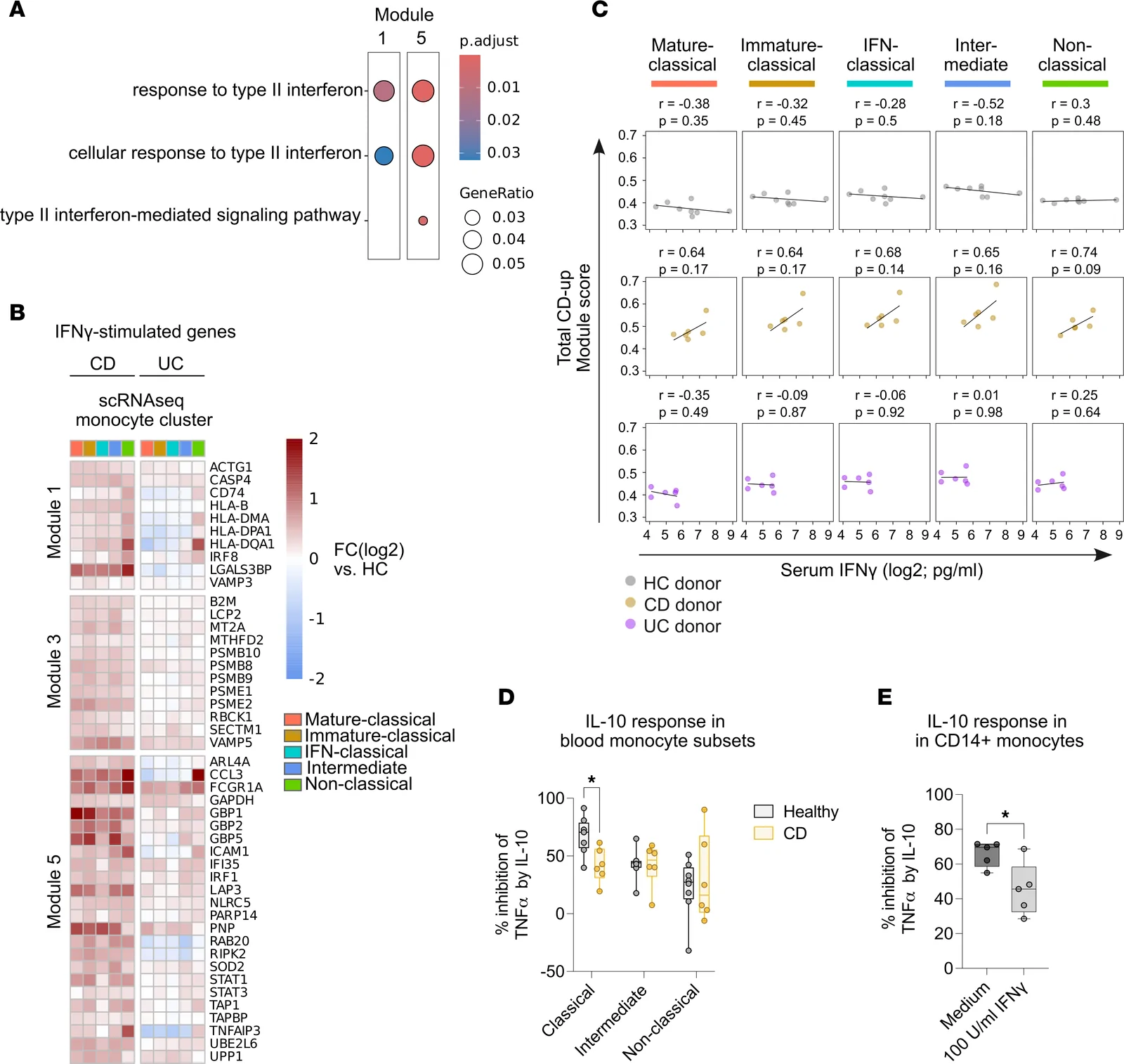
CD monocytes are primed for inflammatory function before they arrive in the intestine, with evidence of IFN-γ–associated conditioning. (**A**) Significantly enriched Gene Ontology terms related to IFN-γ signaling in DEG modules identified in Figure 3. (**B**) Expression of IFN-γ–stimulated genes that are also DEGs is displayed as log_2_ FC relative to HCs for each disease and monocyte cluster. (**C**) Serum concentration of IFN-γ (log_2_) in patients with CD and UC and HCs plotted against the total CD-up module score per monocyte cluster. Pearson’s correlation coefficients (*r*) and corresponding *P* values are shown for each cluster and patient group. Black lines indicate linear regression fits. (**D**) Percentage inhibition of LPS-induced TNF-α production by IL-10 in each blood monocyte subset in HCs (*n* = 8) and a separate cohort of patients with active CD (*n* = 6). Monocyte subsets and intracellular TNF-α production were assessed using flow cytometry following a 3-hour stimulation of PBMCs with 1 ng/mL LPS in the presence and absence of 2 ng/mL IL-10. Data were analyzed statistically by 2-way ANOVA with Šidák’s correction. (**E**) Percentage inhibition of LPS-induced intracellular TNF-α production by 2 ng/mL IL-10 in isolated blood CD14^+^ monocytes from healthy individuals (*n* = 4) preincubated in medium alone or with IFN-γ (100 U/mL) for 24 hours. Data were analyzed by paired *t* test. (**D** and **E**) Box-and-whisker plots show the median (center line), 25th and 75th percentiles (box), and minimum and maximum values (whiskers). Individual data points are overlaid. *P < 0.05.

We next considered the signals that might be responsible for driving the alterations in circulating monocytes from patients with CD and the functional implications this might have. We noted that IFN-γ response genes such as transcription factors (*STAT1*, *IRF1*, *IRF8*), guanylate-binding proteins (*GBP1*, *GBP2*, *GBP5*), and MHC molecules (*HLA-DQA1*, *HLA-B*) were significantly enriched in modules 1, 3, and 5, which comprise genes upregulated in CD (Figure 5, A and B). Despite this, serum IFN-γ levels were not significantly elevated in patients with CD relative to HCs (Supplemental Figure 6C), suggesting that the CD-associated IFN-γ gene signature is not simply explained by increased circulating cytokine abundance at the time of sampling. Additionally, IFN-γ–induced STAT1 phosphorylation was comparable in CD14^+^ monocytes from patients with CD and HCs (Supplemental Figure 6D), and expression of receptors for both type I and type II interferons was unchanged across monocyte clusters between health and disease (Supplemental Figure 6E), implying no intrinsic differences in proximal IFN-γ signaling capacity. However, we did observe a trend toward higher levels of serum IFN-γ in CD compared with UC (Supplemental Figure 6C). In addition, serum IFN-γ levels positively correlated with the magnitude of CD-associated transcriptional changes in monocytes in CD, although this did not reach statistical significance. No such correlation was observed in UC or HCs (Figure 5C). Together, these findings suggest that, within the context of CD, systemic IFN-γ may contribute to shaping monocyte gene expression, although the heightened IFN-γ transcriptional signature in CD cannot be solely attributed to increased circulating IFN-γ levels.

IFN-γ has been shown to reprogram monocyte transcription and function before bone marrow egress in mice (18) and to suppress IL-10 responsiveness in human monocytes (30). We therefore hypothesized that enhanced IFN-γ–associated signaling in CD monocytes might impair their responsiveness to the immunoregulatory cytokine IL-10. To test this, PBMCs from a separate cohort of CD patients with active disease (Supplemental Data File 1) or HCs were stimulated with LPS in the presence or absence of IL-10 and then analyzed by flow cytometry. LPS-induced intracellular TNF-α production in the absence of IL-10 was comparable between patients with CD and HCs across all monocyte subsets (Supplemental Figure 7, A–C); however, IL-10 was significantly less effective at suppressing LPS-induced TNF-α production in classical monocytes from patients with CD (Figure 5D). Importantly, this reduction in IL-10 responsiveness was not attributable to impaired proximal IL-10 receptor signaling, as patients with CD lacked known IBD-associated variants in IL-10 pathway genes (*IL10RA*, *IL10RB*, *STAT3*, *JAK1*, *SOCS3*), exhibited increased surface expression of IL-10Rα, and showed intact IL-10–induced STAT3 phosphorylation (Supplemental Figure 7D). Finally, pretreatment of CD14^+^ monocytes from healthy volunteers with recombinant IFN-γ reduced the ability of IL-10 to suppress LPS-induced TNF-α (Figure 5E). Taken together these data indicate that circulating monocytes in CD are functionally primed toward inflammatory responses before tissue entry, and support a model in which IFN-γ may contribute to imprinting this phenotype.

### The transcriptional and functional reprogramming of CD blood monocytes is maintained upon entry into the intestine.

Having established that circulating monocytes in patients with active CD are transcriptionally and functionally modified in the circulation, we next determined whether this phenotype is retained upon entry into the intestine.

**Figure 6:**
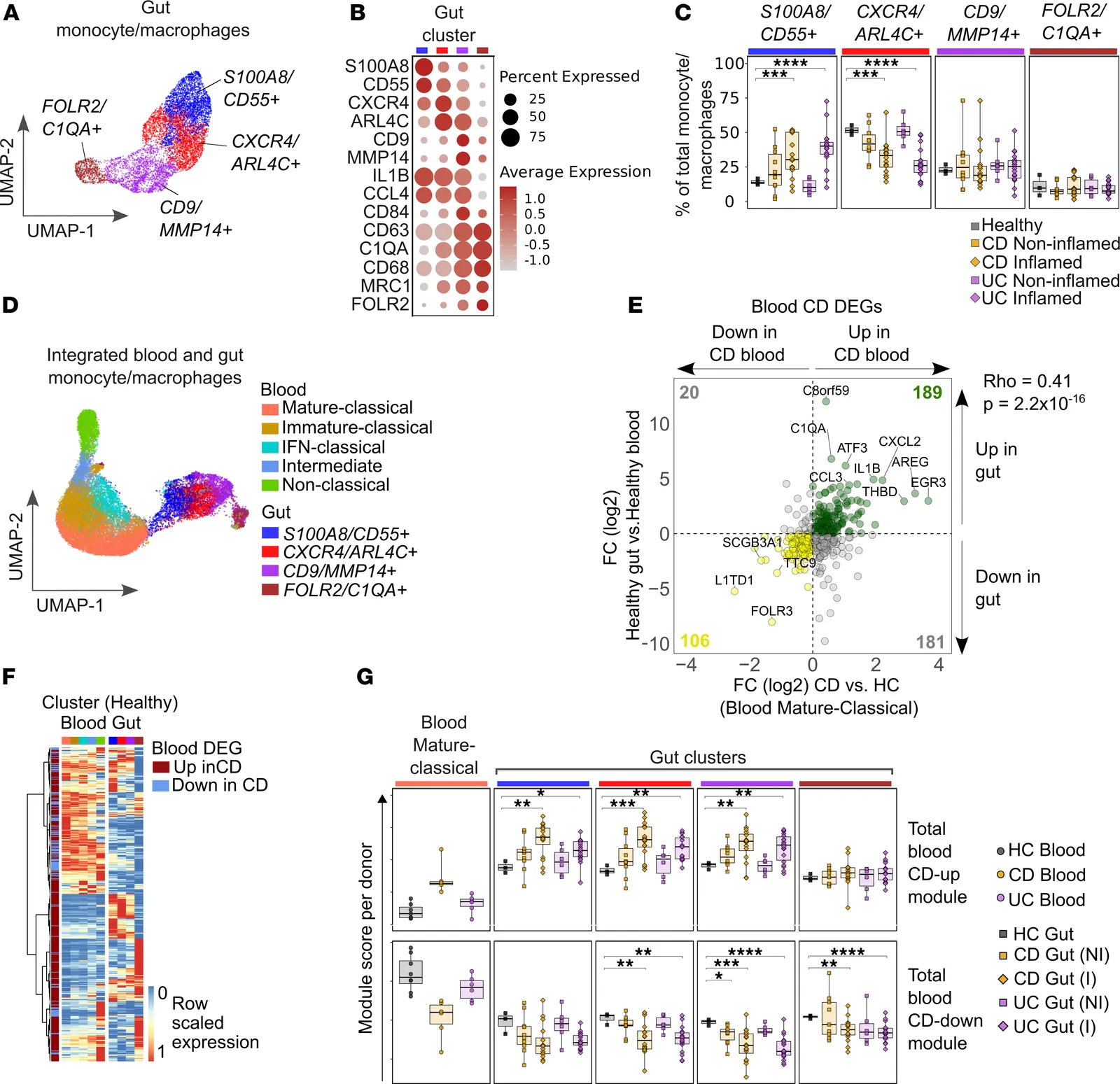
The transcriptional reprogramming of CD blood monocytes is maintained upon entry into the intestine. (**A**) UMAP projection of scRNA-seq data from intestinal monocytes/macrophages derived from inflamed and noninflamed biopsies of biologic-naive patients with CD (*n* = 16) and UC (*n* = 19) and HCs (*n* = 3). Transcriptionally distinct gut clusters are shown. (**B**) Expression of select markers in gut clusters; percent positive cells and log-normalized average expression are shown. (**C**) Proportions of each gut cluster expressed as a percentage of total monocytes/macrophages per donor. *P* value denotes the result of 2-way ANOVA with Dunnett’s correction. (**D**) UMAP projection of integrated intestinal and blood scRNA-seq datasets. Original clusters of independently analyzed datasets are shown. (**E**) Blood CD DEGs plotted as log_2_ FC expression in CD versus HCs in blood mature classical monocytes against log_2_ FC expression in total healthy gut cells versus total healthy blood cells. Numbers of genes appearing in each quadrant and results of Spearman’s rank test are displayed. (**F**) Average expression of blood CD DEGs in blood and gut clusters derived from HCs. Direction of regulation relative to HCs is indicated. (**G**) Total blood CD-up and CD-down module scores plotted per donor in gut monocyte clusters and blood mature classical cells. NI, noninflamed; I, inflamed. *P* value denotes the result of a mixed-effects analysis with Dunnett’s correction within the gut clusters only. (**C** and **G**) Box-and-whisker plots show the median (center line), 25th and 75th percentiles (box), and minimum and maximum values (whiskers). Individual data points are overlaid. *P < 0.05, **P < 0.01, ***P < 0.001, ****P < 0.0001.

To address this, we examined an external myeloid scRNA-seq dataset (31) derived from inflamed and noninflamed intestinal biopsies of biologic-naive patients with IBD (CD, *n* = 16; UC, *n* = 19) and HCs (*n* = 3) (Zenodo, record 14007626). After exclusion of cells with high expression of DC markers (*CD1C*, *CLEC10A*, *LAMP3*, *XCR1*, *IL3RA*) and mast cells (*GATA2*, *CPA3*), the remaining 7,725 cells were segregated into 4 transcriptionally distinct clusters (Figure 6, A and B, and Supplemental Figure 8A).

The *S100A8*/*CD55^+^* cluster, characterized by high expression of *S100A8* and *CD55*, likely represents recently recruited monocytes (3, 32). The *FOLR2*/*C1QA^+^* cluster expressed canonical tissue-resident macrophage markers, including *CD63*, *C1QA*, *CD68*, *MRC1*, and *FOLR2*. The *CD9*/*MMP14^+^* cluster also expressed tissue macrophage markers but was distinguished by increased expression of activation and tissue remodeling genes (*CD9*, *MMP14*, *IL1B*, *CCL4*, *CD84*). Finally, the *CXCR4*/*ARL4C^+^* cluster exhibited intermediate expression of monocyte and macrophage markers, consistent with early-differentiating monocyte-derived cells (Figure 6, A and B, and Supplemental Figure 8B).

There was an expansion of the *S100A8*/*CD55^+^* cluster in inflamed IBD tissue, consistent with previous reports (5–8, 33–36), accompanied by a reduction in the *CXCR4*/*ARL4C^+^* cluster (Figure 6C). Integration with our blood dataset identified the *S100A8*/*CD55^+^* gut cluster as most similar to putatively mature classical blood monocytes based on proximity in uniform manifold approximation and projection (UMAP) space (Figure 6D). This supports the notion that mature classical blood monocytes are precursors to intestinal monocyte-derived populations.

We next examined the expression of blood monocyte CD-associated DEGs within intestinal monocyte/macrophage populations. Firstly, we found that these DEGs broadly reflected transcriptional features of intestinal monocyte/macrophages. Specifically, FCs of these genes in mature classical blood monocytes from patients with CD relative to HCs were positively correlated with FCs of the same genes between total intestinal monocytes/macrophages and total blood monocytes from HCs (Figure 6, E and F). Notably, genes most strongly upregulated in CD blood monocytes (e.g., *EGR3*, *AREG*, *THBD*, and *CXCL2*) were also highly enriched in intestinal compared with blood monocytes. Although this relationship was less pronounced among genes with smaller increases in CD blood monocytes, the overall pattern suggests that blood monocytes in CD acquire some gut-associated transcriptional features before tissue entry.

Next, to directly test whether the blood monocyte CD-associated transcriptional program is preserved in intestinal monocyte/macrophages, we calculated module scores for genes upregulated (CD-up) and downregulated (CD-down) in CD blood monocytes. Intestinal monocytes/macrophages from IBD samples exhibited increased expression of the CD-up module and decreased expression of the CD-down module relative to HCs, recapitulating the pattern originally observed in blood (Figure 6G). This effect was strongest and statistically significant in inflamed CD and UC tissue, although a trend was also evident in noninflamed samples. Interestingly, the CD-up module remained unchanged in *FOLR2*/*C1QA^+^* tissue-resident macrophages. These findings indicate that the blood CD-associated transcriptional signature is preserved in intestinal monocytes/macrophages and is most prominent in inflamed tissue, consistent with its reinforcement by local inflammatory signals.

Similar results were observed in an independent scRNA-seq dataset (37) derived from involved (*n* = 5) and noninvolved (*n* = 4) regions of ileocecal surgical resections from 5 patients with CD (Supplemental Data File 1). In this dataset, *S100A8/9/12^+^* and *C1QA/B/C^+^* monocyte/macrophage clusters showed increased expression of CD-up genes and reduced expression of CD-down genes relative to circulating mature classical monocytes, with the strongest effect occurring in cells from involved tissue (Supplemental Figure 8, C–E). Together, these data suggest a broad pattern whereby the blood CD-associated monocyte signature reflects the premature acquisition of a gut-associated transcriptional profile before tissue infiltration, and that this profile is subsequently amplified within the inflamed intestinal mucosa.

**Figure 7:**
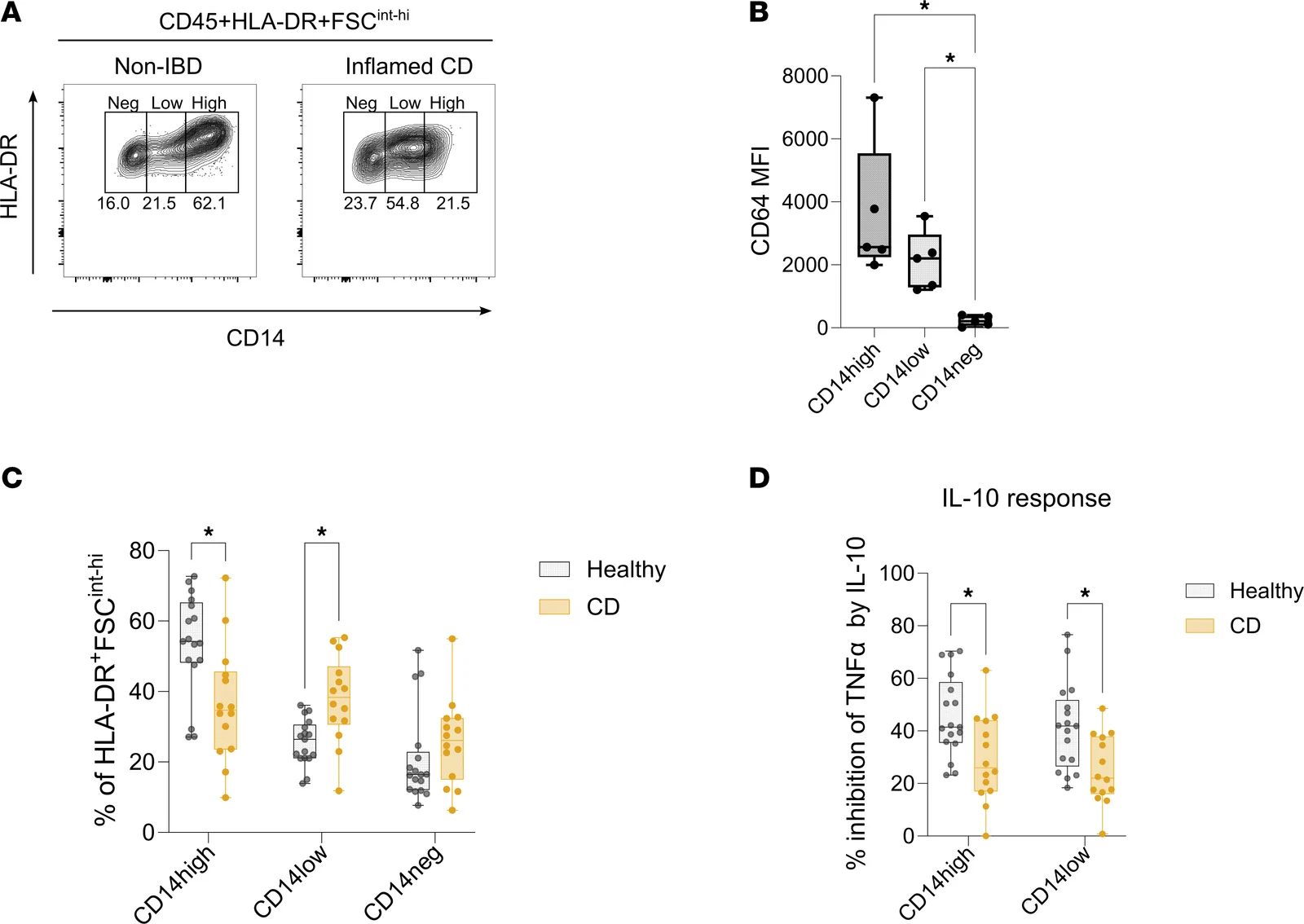
Functional modification of intestinal monocyte-derived cells in CD. (**A**) Flow cytometry gating of CD14^hi^, CD14^lo^, and CD14^neg^ cells within the CD45^+^HLA-DR^+^FSC^int–hi^ population of lamina propria cells (LPCs). Example from inflamed CD and from a non-IBD control are shown. (**B**) Mean fluorescence intensity (MFI) of CD64 (minus MFI of isotype control) in CD14^hi^, CD14^lo^, and CD14^neg^ cells from non-IBD samples (*n* = 5). *P* value denotes the result of 1-way ANOVA with Holm-Šidák’s correction. (**C**) CD14^hi^, CD14^lo^, and CD14^neg^ populations expressed as a proportion of HLA-DR^+^FSC^int–hi^ LPCs from inflamed CD (*n* = 14) and non-IBD controls (Healthy; *n* = 17). (**D**) Percentage inhibition of LPS-induced intracellular TNF-α production by IL-10 in CD14^hi^ and CD14^lo^ LPCs from inflamed CD (*n* = 14) and non-IBD control biopsies (Healthy; *n* = 17). LPCs were stimulated for 3 hours with 1 ng/mL LPS in the presence and absence of 2 ng/mL IL-10. (**C** and **D**) Data were analyzed statistically by 2-way ANOVA with Šidák’s correction. (**B**–**D**) *P < 0.05. Box-and-whisker plots show the median (center line), 25th and 75th percentiles (box), and minimum and maximum values (whiskers). Individual data points are overlaid.

Finally, we addressed whether the functional priming of CD blood monocytes is retained in intestinal monocyte-derived cells. Lamina propria cells (LPCs) were isolated from inflamed intestinal biopsies of patients with CD (Supplemental Data File 1) or from non-IBD tissue and stimulated for 3 hours with LPS in the presence or absence of IL-10 before flow cytometry analysis. Within CD45^+^ leukocytes, three HLA-DR^+^FSC^int–hi^ populations were identified on the basis of CD14 expression: CD14^hi^, CD14^lo^, and CD14^neg^ (Figure 7A and Supplemental Figure 9A). CD14^hi^ and CD14^lo^ populations expressed CD64 consistent with monocyte/macrophage identity, whereas CD14^neg^ cells lacked expression of CD64 and may have included DCs (38) (Figure 7B). CD14^hi^ cells are likely to be a more differentiated macrophage population than CD14^lo^ cells based on higher phagocytic activity, lower CCR2 expression, and higher levels of CX_3_CR1, CD206 (MRC1), CD63, and FOLR2 (Supplemental Figure 9, B–D). There was a relative accumulation of CD14^lo^ cells and corresponding reduction in CD14^hi^ cells in inflamed intestinal mucosa of patients with active CD compared with non-IBD controls (Figure 7, A and C). Although “spontaneous” and LPS-induced production of TNF-α did not differ between groups (Supplemental Figure 9E), IL-10–mediated suppression of TNF-α was significantly impaired in both CD14^hi^ and CD14^lo^ cells from patients with active CD (Figure 7D). Collectively these data indicate that intestinal monocyte-derived cells in CD are predisposed to inflammatory function, and that this phenotype is established, at least in part, prior to tissue recruitment.

## Discussion

Priming of monocyte progenitors by systemic signals prior to tissue recruitment can direct their fate and function (17, 18). Consistent with this notion, we show that in CD, circulating monocytes are already imprinted with a distinct transcriptional and epigenetic program before entering the intestine. Monocytes from patients with UC exhibit fewer transcriptional alterations, suggesting that changes in CD may be disease specific rather than simply reflecting inflammation. Notably, the monocyte transcriptional signature in CD is retained in intestinal monocyte-derived cells and is most prominent in inflamed tissue, suggesting that the local microenvironment amplifies, rather than initiates, features of monocyte dysregulation. Additionally, we found that LPS-induced TNF-α production is less effectively suppressed in both circulating monocytes and intestinal monocyte-derived cells from patients with CD. Given that IL-10 is essential for limiting inflammatory activity of intestinal monocyte-derived cells (10, 13) and for preventing IBD (14), an impaired IL-10 response in CD monocytes is likely to promote intestinal pathology. Together, these findings support a model in which systemic conditioning, in combination with local tissue cues, shapes monocyte dysregulation in CD.

Previous studies have reported alterations in metabolic (39), inflammatory (40, 41), phagocytic (42), and protein processing (41) pathways in CD monocytes, consistent with the pathways enriched in our scRNA-seq dataset. Notably, the strongest transcriptional changes were observed in the putatively least mature classical monocyte population, raising the possibility that conditioning occurs prior to bone marrow egress. Consistent with prior work demonstrating differences in myeloid cell biology between CD and UC (41, 43, 44), transcriptional changes were more pronounced in CD. Although many CD DEGs showed similar directional changes in UC, this transcriptional signature did not correlate with serum CRP, as it did in CD; and showed minimal overlap with the monocyte signature observed in rheumatoid arthritis (45). Collectively, this suggests that the CD-associated monocyte signature is a disease-specific phenomenon rather than a generic response to inflammation. The correlation between the blood CD-associated signature and serum CRP in CD may point to a role for bacterial translocation as a driver of the phenotype, although this remains to be directly tested in larger studies.

Using the single-nucleus multiome dataset, we identified a set of DNA regions with increased accessibility in CD monocytes that were linked to the expression of a subset of inflammatory CD DEGs. These regions were enriched for NF-κB, EGR, KLF, and AP-1 family member binding motifs, implicating their involvement in regulating this set of inflammatory genes. While direct evidence of enhanced activation and DNA binding of these transcription factors in CD monocytes will be required, these findings are consistent with established roles for NF-κB and AP-1 as central mediators of inflammatory responses (46–48), as well as described roles for EGR and KLF family members in myeloid cell activation (49, 50). The upstream signals driving activation of these transcription factors in circulating monocytes remain to be defined, but likely include microbial products and inflammatory cytokines such as IL-1β, TNF-α, and IFN-γ (48–51).

Upregulation of IFN-γ–response genes in blood monocytes from patients with CD implies a role for IFN-γ in systemic monocyte conditioning. Indeed, elevated IFN-γ levels and IFN-γ–producing cells have been widely reported in CD across multiple compartments (5, 52–56), and experimental models have demonstrated that IFN-γ can program monocyte progenitors toward enhanced inflammatory responsiveness (17, 18, 57, 58). IFN-γ–conditioned monocytes exhibit reduced sensitivity to IL-10 via a STAT1-dependent mechanism (30), aligning with our observation that in vitro conditioning with IFN-γ diminishes IL-10–mediated suppression of TNF-α, and that IL-10 responsiveness is impaired in circulating and intestinal monocyte-derived cells from patients with CD. Although the CD-associated transcriptional profile cannot be simply explained by increased circulating IFN-γ levels alone or altered intrinsic signaling capacity, serum IFN-γ levels correlated with the magnitude of the CD-associated monocyte transcriptional signature in CD patients, suggesting that IFN-γ may contribute to monocyte conditioning in a disease context–dependent manner. While roles for type I and type III IFNs cannot be excluded, the overall pattern of gene expression is more consistent with IFN-γ–driven signaling (Supplemental Data File 2). Nevertheless, complex interactions between type I and type II interferons exist (59) and should be explored further in the context of CD.

A linear developmental relationship among circulating human monocytes has previously been described, in which bone marrow–derived classical monocytes either give rise to nonclassical monocytes through an intermediate stage, or disappear by death or migration into tissue (20). Consistent with this model, pseudotime ordering suggested a trajectory (lineage 1) originating from cluster 1 and progressing toward nonclassical monocytes. Based on its transcriptional profile and proximity to HSCs in the pseudotime ordering, we propose that cluster 1 represents the most immature classical monocyte state, although this will require experimental validation. We found no evidence to suggest that the classical monocyte clusters (clusters 0, 1, or 3) correspond to cells of granulocyte-monocyte progenitor or monocyte–dendritic cell progenitor origin as recently identified in mice (60) (data not shown). Importantly, CD was associated with an increased representation of a trajectory arising from the putative immature classical monocyte population and leading toward more mature classical monocytes. This lineage transiently passed through a monocyte state characterized by expression of type I interferon–stimulated genes such as *MX1* and *SIGLEC1* (*CD169*), a population that has been reported in the context of viral infection and inflammatory conditions (61–66) and associated with enhanced CD8^+^ T cell activation capacity (63). In parallel to alterations in these inferred developmental trajectories, we observed that circulating monocytes exhibit a transcriptional profile consistent with premature acquisition of gut-associated features. Together, these findings point to altered monocyte maturation prior to intestinal infiltration in CD, potentially reflecting dysregulated myelopoiesis (15) and/or systemic immune training (16). Further experimental work will be required to delineate these observations and define the mechanisms underlying them.

Monocyte-derived cells in the human intestinal mucosa include both recently recruited *S100A8/A9^+^* monocytes and longer-lived *C1QA/B/C^+^* tissue-resident macrophages (3, 32) consistent with the 4 monocyte/macrophage clusters we identified in an external intestinal dataset showing graded expression of *S100A8* and *C1QA*. Although embryonically derived macrophages may be present in small numbers (32), evidence from a transplantation study in the small intestine concluded that these populations eventually become replaced by circulating precursors over the course of 1 year. This suggests that the majority of cells we classify as monocytes/macrophages in the intestinal dataset are likely derivatives of adult blood monocytes. Integration of the blood and intestinal datasets showed that intestinal *S100A8/CD55^+^* monocytes most closely resemble mature blood classical monocytes (cluster 0), which is suggestive that the latter represent precursors to the intestinal populations. This is consistent with the current understanding that intestinal macrophage populations are replenished by classical monocytes (2, 3, 6, 21); however, we cannot rule out contributions by other blood monocyte populations. Importantly, the transcriptional and functional features observed in circulating CD monocytes were retained in intestinal monocyte-derived populations. This is suggestive that monocytes enter the tissue already biased toward an inflammatory state, rather than being instructed solely by the intestinal microenvironment (11). The blood CD transcriptional profile was also elevated to some extent in monocyte-derived cells from inflamed UC tissue, suggesting that elements of intestinal monocyte dysfunction can occur independently of overt systemic conditioning. Therefore, further work will be required to establish whether monocyte conditioning associates with specific phenotypical features of CD and contributes to disease heterogeneity.

These findings should be interpreted in light of several limitations. The sample size of the single-cell analyses is small, and independent validation of DEGs and inferred differentiation trajectories will be important. In addition, although key aspects of the study were performed in treatment-naive patients, some functional analyses were conducted in individuals receiving therapy, which may introduce confounding effects. While our data support a role for IFN-γ in monocyte conditioning, they do not establish a causal link between IFN-γ signaling and impaired IL-10 responsiveness in vivo; therefore, further mechanistic studies will be required. Finally, the IL-10 response assay was performed using PBMC cultures rather than purified monocytes, which may allow contributions from other immune cell populations.

Overall, our data show that bone marrow–derived circulating monocytes are systemically conditioned in CD, predisposing them toward inflammatory function. Understanding how this imprinting relates to disease heterogeneity and defining the signals that drive it may open new avenues for therapeutic intervention.

## Methods

### Sex as a biological variable.

The study was conducted using samples from both female and male donors. Sex was not considered as a biological variable because of the small sample size.

### Cell preparation.

PBMCs were obtained by centrifugation in Ficoll-Paque PLUS (GE Healthcare). Total and CD14^+^ monocytes were isolated from PBMCs using negative selection (Pan Monocyte Isolation Kit, Miltenyi Biotec) and positive selection (CD14 MicroBeads, Miltenyi Biotec), respectively. Intestinal biopsies were treated with 1 mM dithiothreitol (MilliporeSigma) followed by 1 mM EDTA (MilliporeSigma) in Hanks balanced salt solution (Gibco, Thermo Fisher Scientific) to remove the epithelial layer. The remaining tissue was digested with 1 mg/mL collagenase D (MilliporeSigma) in RPMI 1640 medium (HEPES modification, MilliporeSigma) containing 2% FBS (Gibco, Thermo Fisher Scientific) and 20 μg/mL deoxyribonuclease I (MilliporeSigma). The resultant lamina propria cell (LPC) preparation was passed through a 40 μm strainer before culture and phenotyping.

### scRNA library preparation and sequencing of blood monocytes.

Single-cell libraries were generated from freshly isolated total monocytes using the Chromium Single Cell 3′ Gel Beads-in-Emulsion (GEM), Library and Gel Bead Kit v3 (PN-1000092), as described in the manufacturer’s user guide (10x Genomics).cDNA amplification was performed on the purified GEM–reverse transcription (RT) product, and cDNA was examined for quality using the Agilent 4200 Tapestation with the High Sensitivity D5000 ScreenTape and Reagents (Agilent Technologies) and the Qubit 4.0 Fluorometer and Qubit dsDNA HS Assay Kit (Life Technologies, Thermo Fisher Scientific). Resulting libraries were quantified using the Qubit 4.0 Fluorometer and Qubit dsDNA HS Assay Kit and average fragment size checked using the Agilent D1000 ScreenTape and Reagents. Sequencing was performed on NovaSeq 6000 S4 flow cell (Illumina) with a 150/8/150 read configuration to generate 50,000 reads per cell.

### Single-nucleus multiome library preparation and sequencing of blood monocytes.

Single-nucleus libraries were generated from freshly isolated CD14^+^ monocytes using the Chromium Next GEM Single Cell Multiome ATAC plus Gene Expression Reagent Bundle (PN-1000285) as described in the manufacturer’s user guide (10x Genomics). For the gene expression portion, cDNA amplification was performed on the purified GEM-RT product, and cDNA was examined for quality using the Agilent 4200 Tapestation with the High Sensitivity D5000 ScreenTape and Reagents (Agilent Technologies) and the Qubit 4.0 Fluorometer and Qubit dsDNA HS Assay Kit (Life Technologies, Thermo Fisher Scientific). For the ATAC portion, libraries were generated from the purified GEM-RT product. Both library types were quantified using the Qubit 4.0 Fluorometer and Qubit dsDNA HS Assay Kit and average fragment size checked using the Agilent D1000 (gene expression) and D5000 (ATAC) ScreenTape and Reagents. Sequencing was performed on NextSeq 2000 P3 flow cell (Illumina) with a 150/8/150 read configuration to generate at least 50,000 gene expression reads, and 50,000 ATAC reads per nuclei.

### scRNA-seq data analysis of blood monocytes.

Demultiplexing, barcoded processing, gene counting, and aggregation were performed using the 10x Genomics Cell Ranger pipeline (v6.0.2) (67). Downstream analysis of the resultant expression matrices was performed using R (v4.2.0 and v4.2.2) and Seurat (v4.2.0 and v4.4.0) (68, 69). Seurat objects were generated for each sample (CD, *n* = 6; UC, *n*= 6; healthy, *n* = 8), removing genes expressed in fewer than 3 cells. Samples were then combined, and cells with mitochondrial percentages greater than 25 and unique feature counts of less than 1,000 and greater than 5,000 were filtered out. We performed log normalization and identification of 2,000 variable genes and integrated samples by canonical correlation analysis (CCA) using SelectIntegrationFeatures, FindIntegrationAnchors, and IntegrateData. This was followed by data scaling, principal component analysis (PCA), and preliminary graph-based clustering using FindNeighbors and FindClusters. Clusters expressing *CD14* and/or *FCGR3A* that lacked notable expression of *CD34*, *CD1C*, *LILRA4*, *CD3E*, *PPBP*, *TRDC*, *FCER1A*, *MS4A1*, *TRAC*, and *NKG7* were selected. A *CD34*-expressing cluster was retained for Slingshot analysis. Variable gene identification, data scaling, PCA, and graph-based clustering at a resolution of 0.2 were then recomputed. After removal of an additional minor cluster defined by expression of T cell markers including *IL7R* and *CD3E*, the resulting object contained 19,967 cells. Uniform manifold approximation and projection (UMAP) coordinates were plotted for visualizations. Unique cluster-defining markers were identified using FindAllMarkers, selecting only positive markers with a log_2_ FC cutoff and minimum fraction expression of 0.25 and adjusted *P* value less than 0.05. Where genes were detected multiple times across clusters, the instance with the highest log_2_ FC was retained. Trajectory analysis was performed using Slingshot (v2.6.0) (27). Pseudobulk differential expression analysis was performed using aggregateBioVar (v1.8.0) and DESeq2 (v1.38.3) (70).

### scRNA-seq data analysis of intestinal monocytes/macrophages (external dataset).

Analysis of the external intestinal dataset (Zenodo, record 14007626) was performed using R (v4.2.2) and Seurat (v5.1.0) (71). The filtered myeloid dataset was downloaded in.h5ad format and converted into a Seurat object, excluding genes expressed in fewer than 3 cells and all post-treatment samples. Log-normalization, identification of 2,000 variable genes, data scaling, and PCA were performed. Individual samples, grouped by patient and inflammation status, were integrated by CCA via IntegrateLayers, with k.weight set to 40 and PCA dimensions set to 15. Preliminary graph-based clustering was then performed using FindNeighbors and FindClusters. Clusters with high expression of monocyte/macrophage markers (*CSF1R*, *CD14*, *CD68*, *C1QA*, or *S100A8*) and low expression of DC markers (*CD1C*, *CLEC10A*, *LAMP3*, *XCR1*, *IL3RA*) and mast cells (*GATA2*, *CPA3*) were selected. After removal of samples containing fewer than 30 cells, variable gene identification, data scaling, PCA, CCA integration, and graph-based clustering at a resolution of 0.2 were recomputed. UMAP coordinates were plotted for visualizations. The resulting object contained 7,725 total cells derived from 5 different gut regions (ascending colon, descending colon, rectum, sigmoid, and terminal ileum) and from patients with CD (*n* = 16) and UC (*n* = 21) and HCs (*n* = 3). Cells from patients with IBD were derived from both inflamed and noninflamed tissue (Supplemental Figure 8A). Unique cluster-defining markers were identified using FindAllMarkers, selecting only positive markers with a log_2_ FC cutoff and minimum fraction expression of 0.25 and adjusted *P* value less than 0.05. Where genes were detected multiple times across clusters, the instance with the highest log_2_ FC was retained.

### Integration of blood and intestinal scRNA-seq datasets.

Integration of the blood and the external intestinal dataset was performed using R (v4.2.2) and Seurat (v5.1.0) (71). log normalization, identification of 2,000 variable genes, data scaling, and PCA were performed on combined cells from the blood and intestinal datasets derived as outlined above. Harmony integration was then performed using IntegrateLayers with theta set to 6. UMAP coordinates were plotted for visualizations.

### Single-nucleus multiome data analysis of blood monocytes.

Cell Ranger ARC (v2.0.0) was used for demultiplexing base call files (BCLs) from gene expression and ATAC-sequenced libraries using cellranger-arc mkfastq, followed by cellranger-arc count for genome alignment, gene expression quantification, and ATAC peak calling. Downstream analysis was then performed using R (v4.2.2), Seurat (v5.1.0) (71), and Signac (v1.13.0) (72) in line with the package vignettes. Seurat objects containing gene expression (RNA) and chromatin (ATAC) assays were generated for each of the 6 CD14^+^ peripheral blood monocyte samples (CD, *n* = 3; healthy, *n* = 3), which were then combined using *merge*. Intersecting peaks were merged using *reduce*, and the unified peak list was recounted using *FeatureMatrix*. Peaks with widths less than 20 or greater than 10,000 nucleotides as well as those on nonstandard chromosomes were removed. Further subsetting was then performed by filtering out of cells with mitochondrial percentages greater than 30; unique RNA counts less than 500 and greater than 7,500; unique ATAC counts less than 1,000 and greater than 30,000; transcription start site enrichments less than 1; and nucleosome signals greater than 2. For the RNA assay, log-normalization, identification of 2,000 variable genes, data scaling, and PCA were computed before sample integration by CCA using *IntegrateLayers* with k.weight set to 81. Preliminary graph-based clustering was performed, and the object was further subsetted to exclude minor clusters of cells with notable gene expression of dendritic cell (*CD1C*, *CLEC10A*), T cell (*CD247*), NK cell (*NKG7*), and B cell (*MS4A1*) markers, yielding a monocyte object of 5,892 single nuclei. Graph-based clustering was then recomputed at a resolution of 0.5. For the ATAC assay, term frequency–inverse document frequency normalization followed by singular value decomposition on all peaks of the monocyte object was computed. Samples were then integrated by reciprocal latent semantic indexing LSI (rlsi) using FindIntegrationAnchors and IntegrateEmbeddings with k.weight set to 39, and then graph-based clustering was performed at a resolution of 0.3. UMAP coordinates were plotted for visualizations. Unique RNA cluster–defining markers were identified using FindAllMarkers, selecting only positive markers with a log_2_ FC cutoff and minimum fraction expression of 0.25 and adjusted *P* value less than 0.05 and setting disease status as a latent variable. Where genes were detected multiple times across clusters, the instance with the highest log_2_ FC was retained for downstream analyses. Differentially accessible peaks (DAPs) between CD and HC cells were identified in each RNA cluster by logistic regression using FindMarkers, selecting features with a minimum fraction accessibility of 0.1, and setting the total number of fragments as a latent variable. Peaks with an adjusted P value less than 0.05 were defined as DAPs. Peak-gene links were identified using LinkPeaks. Transcription factor motif enrichment analysis of motifs retrieved from the JASPAR 2020 database (73) was performed using *FindMotifs*, setting a selection of 50,000 peaks with matched guanine–cytosine content to the peak list of interest as a background list. Nearest genes to unlinked peaks were identified using *ClosestFeature*.

### Pathway enrichment.

Enrichment of Gene Ontology (biological process) terms was achieved using the R package clusterProfiler (v4.18.4) (74). compareCluster was used to determine enriched terms in selected lists of genes. To reduce redundancy among these enriched terms, a semantic similarity–based approach was performed using the simplify function. Terms with a similarity score greater than 0.5 were considered redundant, and among such terms the one with the lowest adjusted *P* value was retained.

### Calculation of gene module scores.

Module scores were calculated with Seurat’s AddModuleScore function using default settings.

### IFN-γ conditioning of CD14^+^ monocytes.

Freshly isolated CD14^+^ monocytes were cultured at 1 × 10^6^ cells per mL in complete medium (Dutch-modified RPMI 1640 medium [MilliporeSigma] containing 10% FBS [Gibco, Thermo Fisher Scientific], 100 U/mL penicillin, 100 μg/mL streptomycin [MilliporeSigma], and 2 mM l-glutamine [MilliporeSigma]) in the presence or absence of recombinant human IFN-γ (Bio-Techne; 100 U/mL) for 24 hours.

### IL-10 response assay.

Freshly isolated PBMCs, LPCs, or CD14^+^ monocytes conditioned with or without IFN-γ were cultured at 1 × 10^6^ to 2 × 10^6^ cells/mL in complete medium as above. Cells were stimulated with 1 ng/mL lLPS (from *E*. *coli* O111:B4; MilliporeSigma) for 3 hours in the presence of 3 μM monensin (Invitrogen, Thermo Fisher Scientific). Two nanograms per milliliter recombinant IL-10 (Bio-Techne) was added for 30 minutes before LPS where required. Fifty micromolar of an ADAM17 inhibitor (TMI-005, Axon Medchem) was included in all PBMC cultures in order to prevent shedding of CD16 upon LPS stimulation (75). Cells were then analyzed by flow cytometry to measure intracellular TNF-α within monocyte populations.

### Flow cytometry.

Staining for surface markers was performed in FACS buffer (Ca^2+^- and Mg^2+^-free PBS containing 2% FBS, 0.02% NaN_3_, and 1 mM EDTA) for 30 minutes on ice with fluorescently labeled antibodies in the presence of an Fc block (BioLegend). Intracellular staining was achieved by fixation and permeabilization of cells using Leucoperm reagents (Bio-Rad) and then staining in FACS buffer on ice for 30 minutes with fluorescently labeled antibodies. For IL-1β staining, PBMCs were stimulated with 0.4 ng/mL LPS (from *E*. *coli* O111:B4; MilliporeSigma) or medium alone for 3 hours in the presence of 50 μM ADAM17 inhibitor (TMI-005, Axon Medchem). For intranuclear staining, PBMCs were stimulated for 15 minutes at 37°C with IFN-γ (Bio-Techne; 100 U/mL) or medium alone, followed by fixation and permeabilization using Cytofix buffer and Phosflow Perm Buffer III (BD Biosciences) and then staining on ice for 30 minutes with fluorescently labeled antibodies. For lists of flow cytometry antibodies, see Supplemental Methods. Samples were acquired on either a BD LSRII or a BD FACSCanto cytometer (BD Biosciences). Data were analyzed using WinList v9.0 (Verity Software House) and FlowJo v8 and v10.

### Statistics.

Details of statistical analysis of scRNA-seq and single-nucleus multiome data are described in the sections above. Other statistical analyses were performed using GraphPad Prism (v10.0) for parametric and nonparametric tests and ANOVA, and R (v4.2.2) for correlation analyses. To test health versus disease effects across different monocyte populations or clusters, 2-way ANOVA was used with Šidák’s correction when comparing 2 groups, or Dunnett’s correction when comparing more than 2 groups. A mixed-effects analysis was used instead of 2-way ANOVA in the case of missing data. To test 3 groups of normally distributed paired data, a 1-way ANOVA with Holm-Šidák correction was used. To test 2 groups of normally distributed data, a paired or unpaired *t* test was used, and 2-sided *P* values are reported. A Kruskal-Wallis test with Dunn’s correction was used to compare 3 groups of non-normally distributed, unpaired data. To assess the linear relationship between IFN-γ expression and module scores, correlation coefficients were calculated with Pearson’s correlation test using the cor.test function, which provides both the correlation coefficient (*r*) and corresponding 2-sided *P* value. To evaluate concordance in gene expression FCs, Spearman’s rank correlation coefficient was calculated using the *cor.test* function.

### Study approval.

Peripheral blood from patients with IBD and all intestinal samples were collected under ethical approvals 15/LO/1230 and 20/LO/1230 from patients at the Royal London Hospital. Peripheral blood from healthy volunteers was collected under ethics approval QMERC2018/93. Resection specimens were obtained from patients with CD undergoing stricture surgery. Intestinal biopsies were taken from patients with CD and control individuals undergoing investigation for altered bowel habit or rectal bleeding with no evidence of IBD. All donors gave informed written consent.

### Data availability.

Peripheral blood scRNA-seq and multiome data are available in the NCBI’s Gene Expression Omnibus (GEO) database (accession numbers GSE268626 and GSE277496, respectively). Intestinal resection scRNA-seq data are available at Array Express (accession number E-MTAB-11792). Values for all data points in graphs are reported in the Supporting Data Values file.

## Author contributions

EH contributed to conceptualization, data curation, formal analysis, investigation, methodology, project administration, visualization, writing of the original draft, review and editing of the manuscript, and funding acquisition. RG contributed to conceptualization, data curation, investigation, methodology, and project administration. IH contributed to data curation, formal analysis, investigation, methodology, and project administration. EW contributed to investigation and writing of the original draft. JRB contributed to formal analysis. EC contributed to investigation. PS contributed to investigation and writing of the original draft. HC contributed to investigation. AL contributed to data curation, investigation, project administration, and resources. AS contributed to project administration, resources, and review and editing of the manuscript. JOL contributed to conceptualization, funding acquisition, supervision, and review and editing of the manuscript. AJS contributed to conceptualization, formal analysis, funding acquisition, supervision, and review and editing of the manuscript.

## Conflict of interest

EH received speaker fees from Gilead and research support from Gilead and AbbVie. RG received research support from Gilead and AbbVie. AL is now employed by and has share options with Engitix. JOL served as a consultant and an advisory board participant for AbbVie, Bristol Myers Squibb, Celgene, Celltrion, Eli Lilly, Engitix, Ferring Pharmaceuticals, Galapagos, Gilead, GSK, Janssen, MSD, Napp, Pfizer, Shire, Takeda, and Vifor Pharma; has received speaker fees and sponsorship for academic meetings from AbbVie, Ferring Pharmaceuticals, Janssen, MSD, Napp, Norgine, Pfizer, Shire, Takeda, and Tillotts Pharma; and has received investigator-led research grants from AbbVie, Gilead, Pfizer, Shire, and Takeda. AJS has received research funding from AbbVie, Gilead, and Takeda.

## Funding support

- Gilead Sciences Inc. (to AJS and JOL).
- The Willoughby Trust (to AJS and JOL).
- Bowel Research UK (to AJS, JOL, and EH).
- Barts Charity (to AJS and JOL).

## Supplementary Material

Supplemental data

Supplemental data set 1

Supplemental data set 2

Supporting data values

## Version 1 08/11/2026

In-Press Preview

## Version 2 09/22/2026

Electronic publication
