# Supplementary material for: Systemic reprogramming of monocytes in Crohn’s disease promotes their intestinal inflammatory function
Source: JCI Insight. 2026 Aug 11;11(18):e192830. doi: 10.1172/jci.insight.192830 (PMC13596727; doi:10.1172/jci.insight.192830)
Supplement: Supplementary file 1 [file jciinsight-11-192830-s102.pdf]

SUPPLEMENTAL MATERIAL

Methods

Single-cell library preparation and sequencing of cells derived from intestinal resection tissue

Full-thickness resection specimens were classified as involved or non-involved based on the presence or absence of fibrotic structures as determined by a gastrointestinal pathologist. Cells were then extracted by an enzymatic extraction protocol (1) and cryopreserved in line with the recommended 10x Genomics protocol for Fresh Frozen Human Peripheral Blood Mononuclear Cells. Single-cell libraries were generated from cryopreserved intestinal cells derived from resected tissue as described for blood monocytes. Sequencing was performed on NextSeq500 (Illumina) with a 26/8/98 read configuration to generate 50,000 reads per cell.

Single-cell RNA sequencing data analysis of intestinal monocyte/macrophages derived from resection tissue

De-multplexing, barcoded processing, gene counting and aggregation were performed using 10x Genomics Cell Ranger pipeline (v3.1.0). Downstream analysis of the resultant expression matrices was performed using R (v4.2.0 & v4.2.2) and Seurat (v4.2.0 & v4.4.0). Seurat objects were generated for each sample (involved n=5; non-involved n=4) removing genes expressed in fewer than 3 cells. Cells with mitochondrial percentages > 25 and unique feature counts < 500 and > 4000 were filtered out. Log-normalization and identification of 2000 variable genes was performed, and samples were integrated by canonical correlation analysis (CCA) using *SelectIntegrationFeatures, FindIntegrationAnchors* and *IntegrateData*, before data scaling, principal components analysis (PCA) and preliminary graph-based clustering. Cells expressing *CD45* and *CD14* and/or *CD16* (log-normalized counts > 0) and lacking expression of *CD1C*, *LILRA4*, *CD3E*, *TRDC*, *FCER1A*, *MS4A1*, *TRAC*, and *NKG7* (log-normalized counts = 0) were selected. Variable gene identification, data scaling, PCA and graph-based clustering at a resolution of 0.2 were then recomputed for each dataset. The resulting intestinal monocyte/macrophage object contained 288 cells.

Gene Set Enrichment Analysis (GSEA)

GSEA analysis was performed on each pseudobulk differential expression analysis using fgsea (v1.24.0). See Supplemental Figure 3 legend for further details.

Phagocytosis assay

LPCs were prepared at $1x10^6$ cells per mL in complete medium (Dutch modified RPMI 1640 medium (Sigma-Aldrich) containing 10% fetal bovine serum (Gibco), 100 U/mL penicillin, 100 µg/mL streptomycin (Sigma-Aldrich) and 2 mM L-Glutamine (Sigma-Aldrich)). Fluoresbrite® YG Carboxylate Microspheres (Polyscience, 2µm) were added at a concentration of $1x10^7$ particles/mL and then incubated at 37°C for 1.5 hours. Control (4°C) samples were chilled on ice for 15 minutes prior to the addition of microspheres and then incubated on ice for an equivalent period of time. Samples were then washed, stained for surface markers as previously described, and then acquired on a BD FACSCanto II cytometer. Data were analysed using WinList v9.0 (Verity Software House).

PhosFlow pSTAT3 staining

PBMCs ($1x10^6$) were stained for surface markers (described previously) and then resuspended in 500 µL FACS buffer before stimulating for 15 minutes at 37ºC with recombinant human IL-10 (100 ng/mL; Bio-techne). 500 µl of 4% paraformaldehyde was added and left for a further 15 minutes. For intracellular staining, cells were permeabilised in 1mL of cold 70% methanol on ice for 30 mins. The cells were then stained for phosphorylated STAT3 (pSTAT3) in FACS buffer on ice for 30 minutes, and finally fixed in 1% paraformaldehyde.

Genotyping

Amplicon libraries targeting five genes (*IL10RA, IL10RB, SOCS3, STAT3* and *JAK1*) were generated from patients with Crohn's disease using the Fluidigm LP 48.48 Array integrated fluidic circuit (IFC). Sequencing was performed using the Illumina MiSeq and data analysis performed using Partek Flow.

Table of antibodies used for flow cytometry

Antigen	Flurochrome	Clone	Isotype	Manufacturer
CD64	APC-Cy7/PE-Cy7	10.1	Mouse IgG1	Biolegend
CD206 (MMR)	FITC/APC	15-2	Mouse IgG1	Biolegend
STAT1	Alexa Fluor 647	1/Stat1 (RUO)	Mouse IgG1	BD Biosciences
CX3CR1	FITC	2A9-1	Rat IgG2b	Biolegend
IL-10RA	PE	3F9	Mouse IgG1	Biolegend
CD16	PerCP-Cy5.5/PE	3G8	Mouse IgG1	Biolegend
pSTAT3 (pY705)	AlexaFluor488	4/P-STAT3	Mouse IgG2A	BD Biosciences
FOLR2	APC	94b/FOLR2	Mouse IgG1	Biolegend
pSTAT1 (pY701)	Alexa Fluor 488	4a (RUO)	Mouse IgG2a	BD Biosciences
IL-1β	FITC	H1b-98	Mouse IgG2b	Biolegend
CD63	APC-Cy7/APC	H5C6	Mouse IgG1	Biolegend
CD14	Pacific Blue/PE-Cy7	HCD14	Mouse IgG1	Biolegend
CD45	PE/ Pacific Blue	HI30	Mouse IgG1	Biolegend
CD55	APC	JS11	Mouse IgG1	Biolegend
CCR2 (CD192)	PerCP-Cy5.5/APC	K036C2	Mouse IgG2A	Biolegend
HLA-DR	APC/PE-Cy7/FITC	L243	Mouse IgG2a	Biolegend
CD14	Pacific Blue	M5E2	Mouse IgG2a	Biolegend
TNFα	PE/PE-Cy7	Mab11	Mouse IgG1	Biolegend
CD64	APC	S18012C	Mouse IgG1	Biolegend
CD87	PE	VIM5	Mouse IgG1	Biolegend

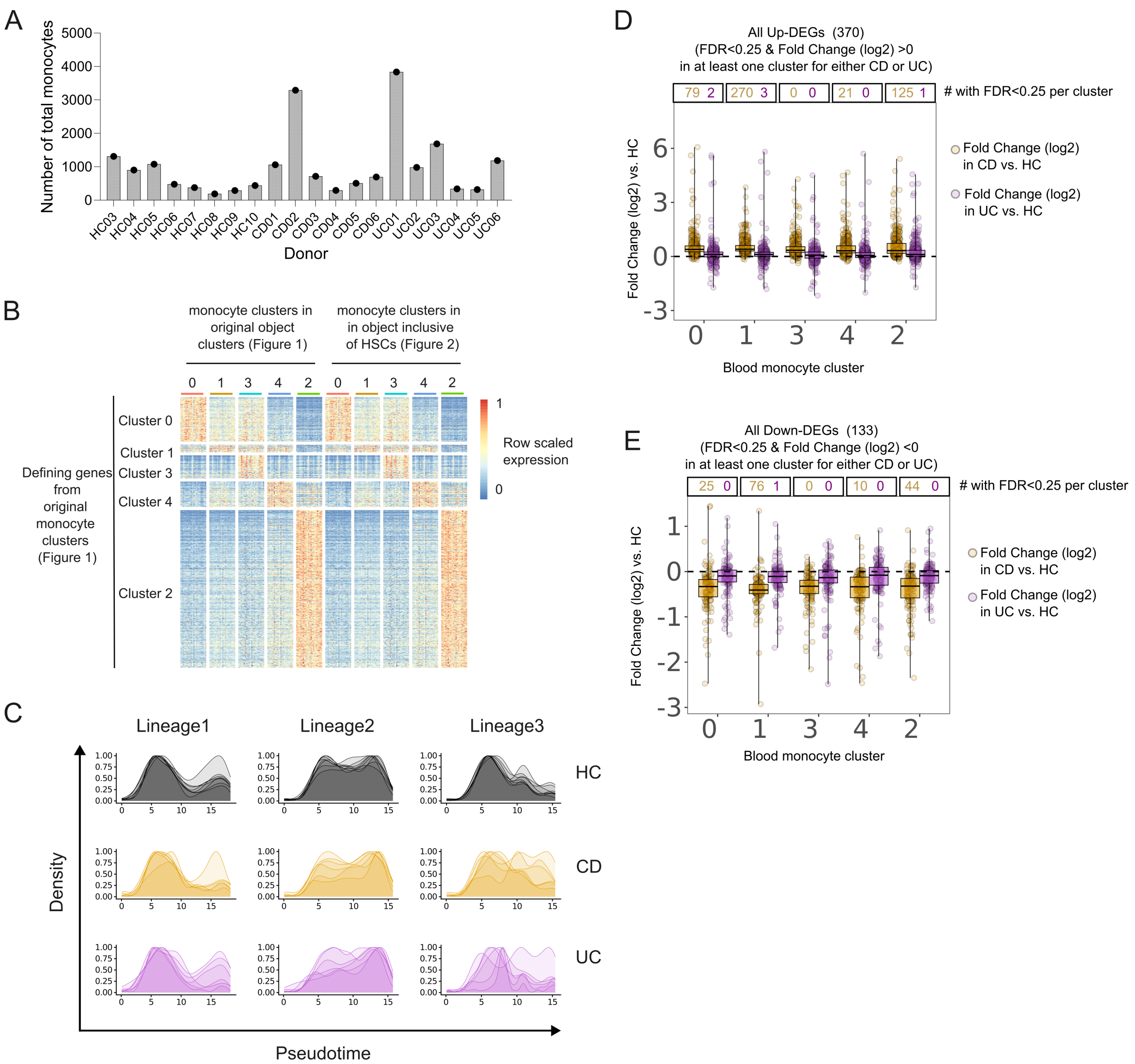

Supplemental Figure 1. scRNA-seq analysis of peripheral blood monocytes from newly diagnosed, treatment-naive IBD patients with active disease (Crohn's disease [CD: n= 6]; ulcerative colitis [UC: n= 6]) and healthy controls (HC; n = 8). **(A)** Number of total monocytes per donor. **(B)** Heatmap displaying cluster-defining markers from Figure 1C. Average cell expression per donor in monocyte clusters from Figure 1 (left panels) and Figure 2 (right panels). **(C)** Density plots showing the distribution of cells across each Slingshot-defined pseudotime lineage per donor. **(D)** All upregulated or **(E)** downregulated DEGs in CD and UC relative to healthy controls (FDR < 0.25 in at least one cluster/disease) plotted as a $\log_2$ fold change (FC) compared to healthy controls in all clusters and both diseases. Number of genes with FDR < 0.25 in each cluster is indicated for each disease. Box-and-whisker plots show the median (center line), the 25th and 75th percentiles (box), and minimum and maximum values (whiskers). Individual observations are overlaid.

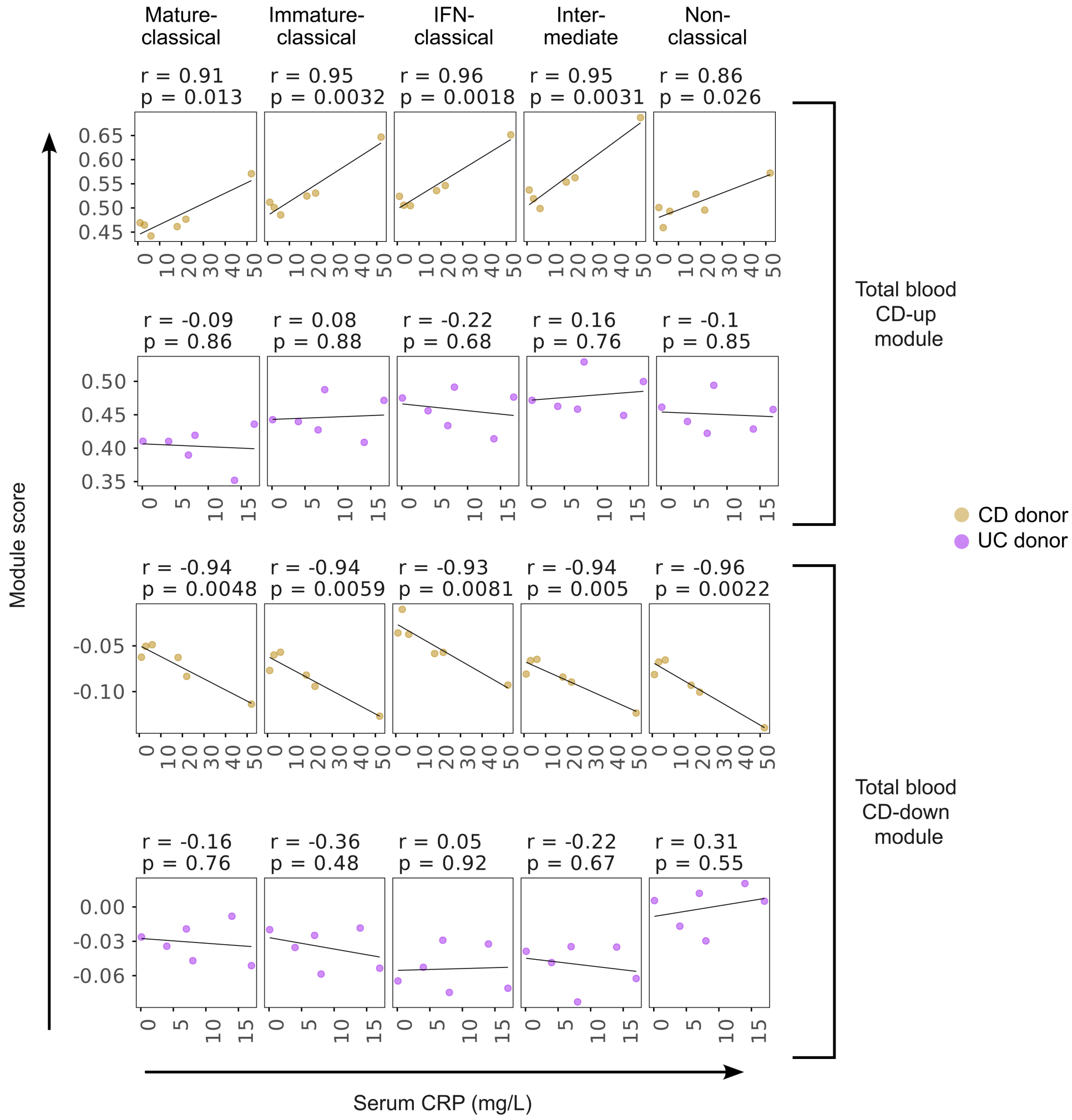

Supplemental Figure 2. Serum concentration of C-reactive protein (CRP) in patients with Crohn's disease (CD) and Ulcerative Colitis (UC) plotted against total CD-Up and CD-Down module scores per monocyte cluster. Pearson correlation coefficients (r) and corresponding *p*-values are shown for each cluster. Black lines indicate linear regression fits.

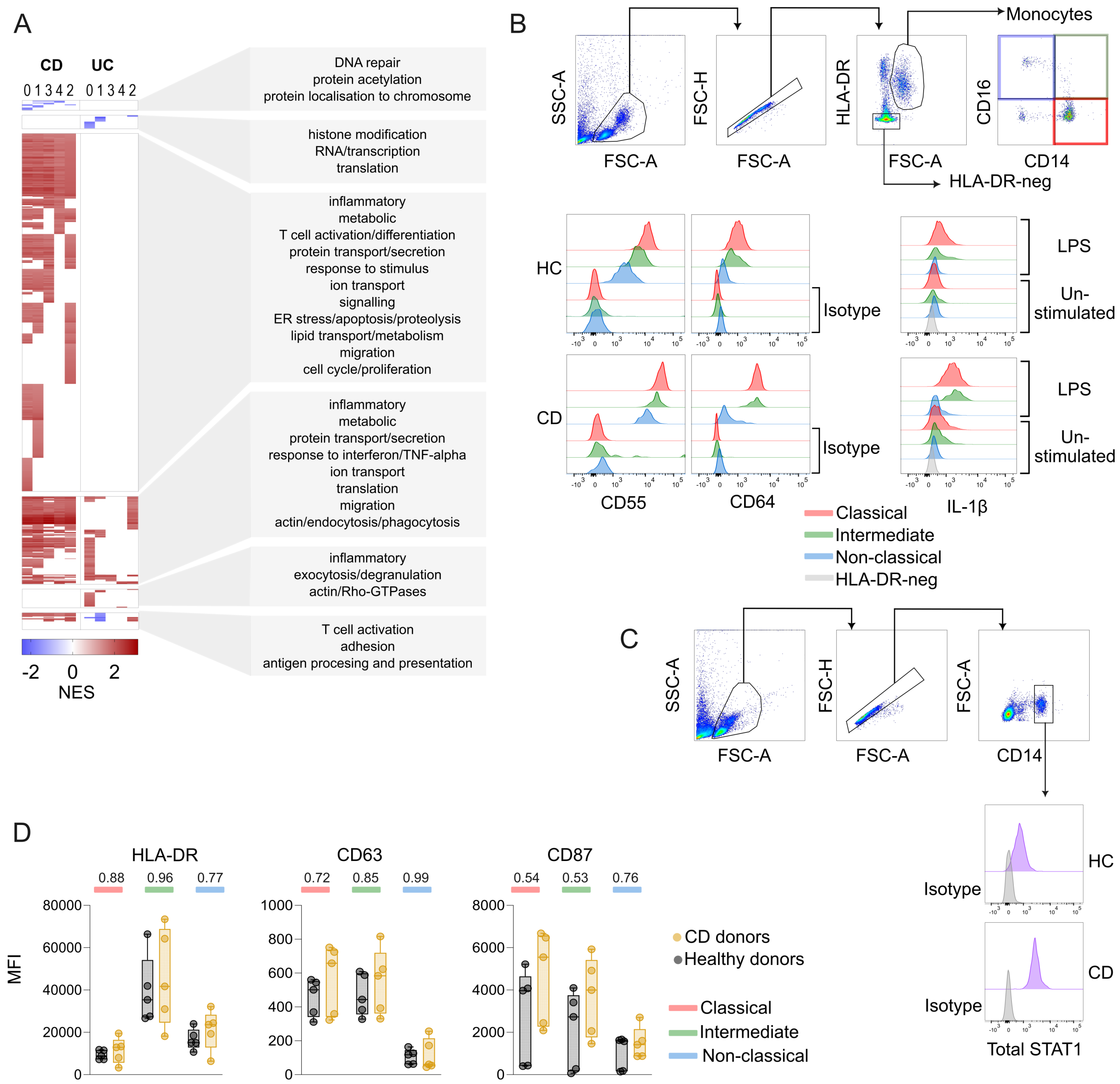

Supplemental Figure 3. (A) Gene Set Enrichment Analysis (GSEA) was performed on each pseudobulk differential analysis described in Figure 3 (one analysis per cluster per disease: ten analyses in total). Net Enrichment Score (NES) of terms from either 'Reactome', 'Gene Ontology-biological process', or 'Hallmark' databases (adjusted p-value < 0.01, NES $> 1.5/< -1.5$, size of term > 15 genes) is shown for each of the ten analyses (819 terms in total). Terms are grouped according to positive or negative enrichment in either Crohn's disease (CD) or ulcerative colitis (UC) or both. Manual annotations are displayed. **(B)** Flow cytometry plots showing extra-cellular CD55 and CD64, and intra-cellular IL-1β in classical, intermediate and non-classical monocytes. For intra-cellular IL-1β staining, cells were first cultured for 3 hours in the presence or absence (unstimulated) of 0.4 ng/mL LPS. **(C)** Flow cytometry plots showing intra-nuclear STAT1 staining in total $CD14^{+}$ monocytes. **(D)** Flow cytometry analysis of monocytes in treatment-naïve patients with active CD (n=5) and healthy controls (n=5). Mean Fluorescence Intensity (MFI) of extra-cellular HLA-DR and CD63 and CD87 (minus MFI of isotype control) in classical, intermediate and non-classical monocytes. p-values denote the result of two-way ANOVA with Šídák's correction. Box-and-whisker plots show the median (center line), the 25th and 75th percentiles (box), and minimum and maximum values (whiskers). Individual observations are overlaid.

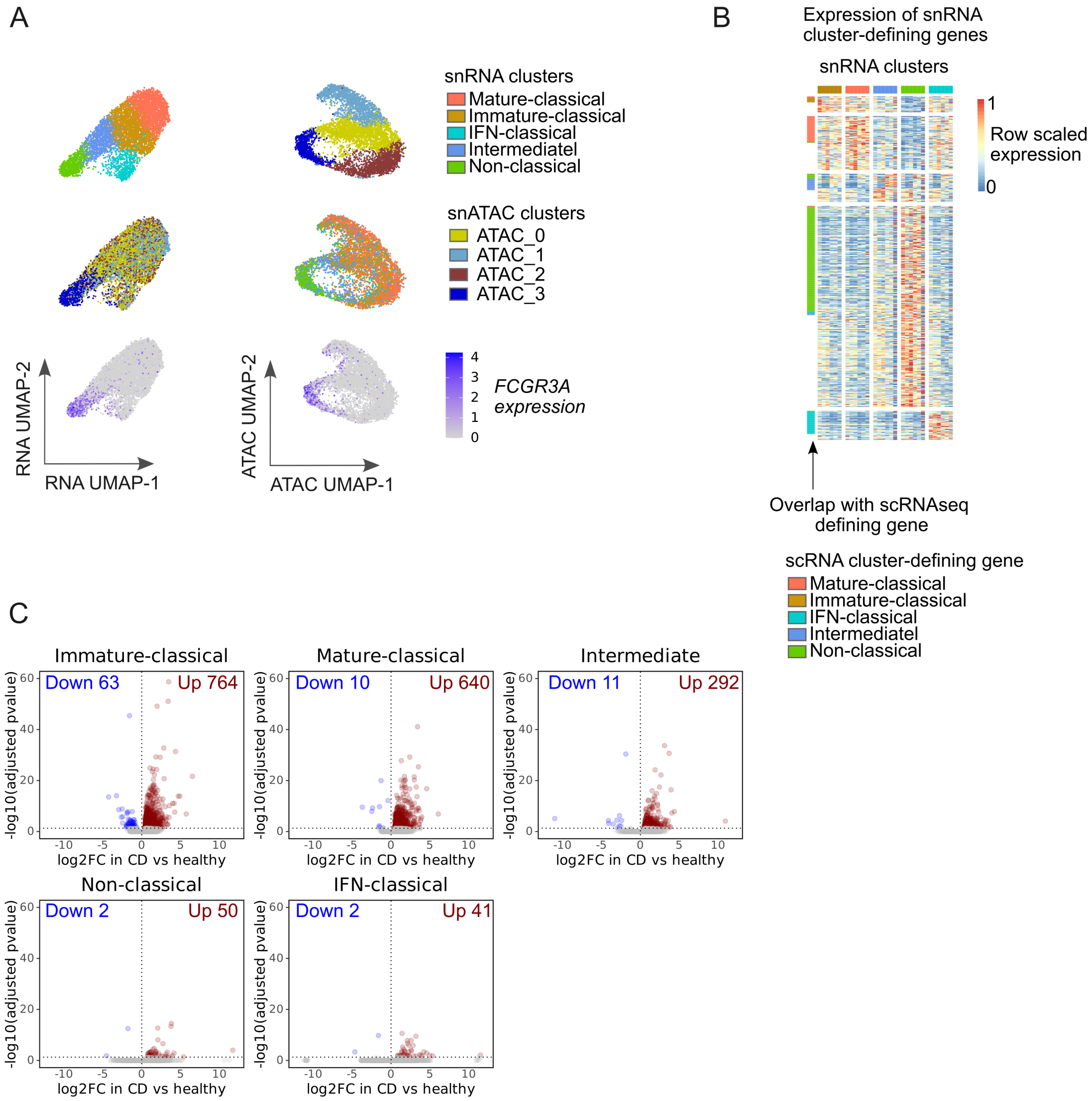

Supplemental Figure 4. Single-nucleus multiomics (snRNA-seq and snATAC-seq) analysis of peripheral blood CD14$^+$ monocytes from treatment-naïve, newly diagnosed active CD patients (CD; n=3) and healthy controls (HC; n=3). **(A)** UMAP projections of snRNA-seq and snATAC-seq monocyte clusters from all integrated donors. Clusters are plotted on both snRNA-seq and snATAC-seq UMAP coordinates as indicated. Log-normalized expression of *FCGR3A* in cells plotted on both snRNA-seq and snATAC-seq UMAP coordinates is shown. **(B)** Average cell expression per donor per cluster of snRNA-seq cluster-defining genes. Overlap with scRNA-seq defining genes is indicated. **(C)** Differential accessibility analysis was performed within each snRNA-seq monocyte cluster to compare cells from treatment-naïve patients with active Crohn's disease (CD; n=3) with cells from healthy controls (HC; n=3). Log$_2$ fold change (FC) accessibility in CD versus health and -log$_{10}$ adjusted *p*-value is shown for peaks within each snRNA-seq monocyte cluster. Number of Differentially Accessible Peaks (DAPs) (adjusted *p*-value < 0.05) for each cluster is indicated.

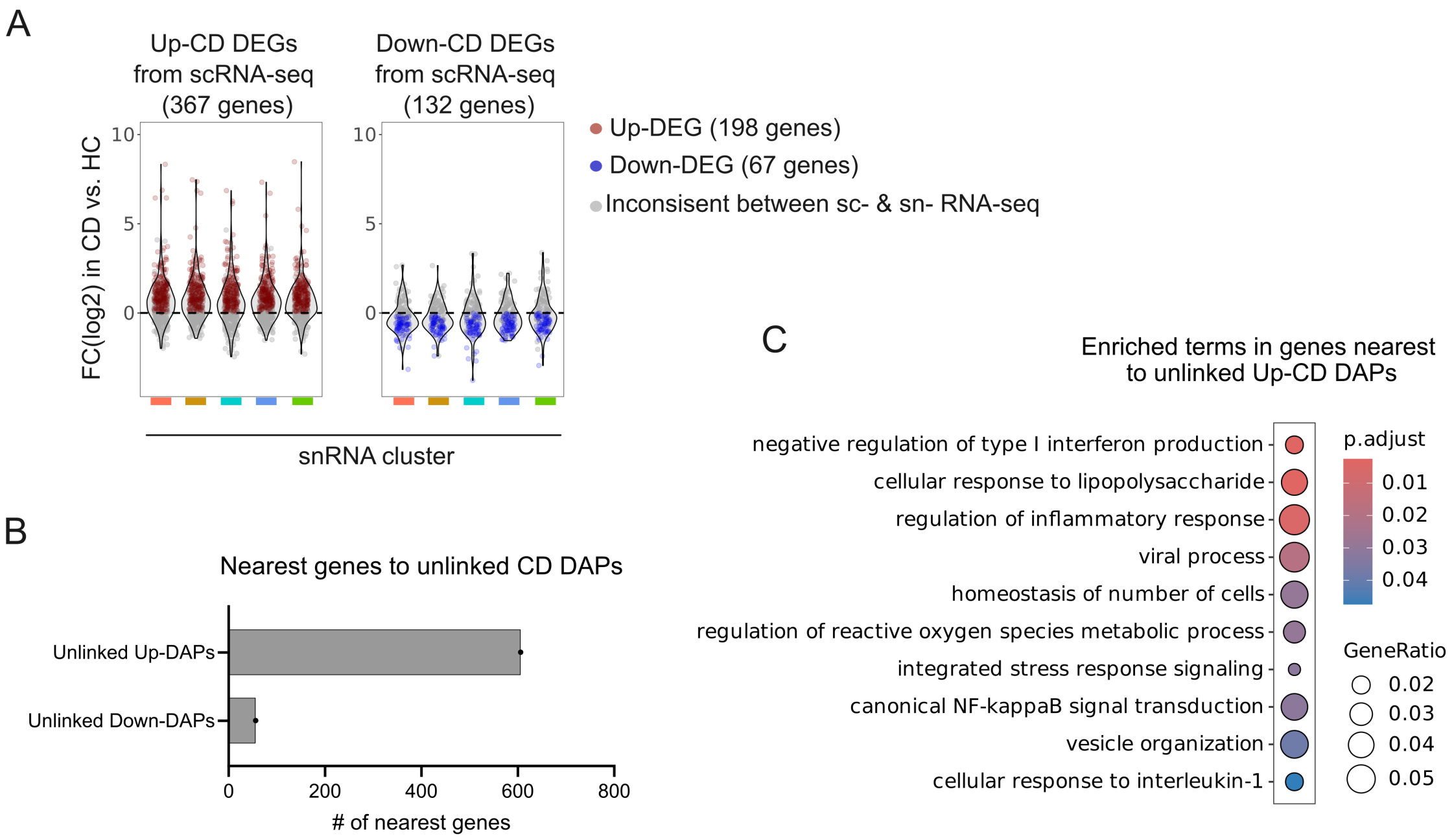

Supplemental Figure 5. (A) Log_2 FC in expression of scRNA-seq CD DEGs (identified in at least one scRNA-seq cluster in Figure 3) plotted in snRNA-seq clusters. Up-DEGs are defined as being scRNA-seq Up-CD DEGs with a positive log_2 FC across all snRNA-seq clusters (198 genes). Down-DEGs are defined as being scRNA-seq Down-CD DEGs with a negative log_2 FC across all snRNA-seq clusters (67 genes). Violin plots show the distribution of log2 FC values (kernel density estimate). Individual observations are overlaid as jittered points. The dashed horizontal line indicates log2 FC = 0. **(B)** Number of nearest genes to Up- and Down- DAPs that are unlinked to gene expression are plotted. **(C)** Top 10 significantly enriched Gene Ontology terms (Biological Process) for genes nearest to Up-CD DAPs that are unlinked to gene expression. Gene ratio, representing the number of genes in the term divided by the total number of defining genes for that cluster, and adjusted *p*-value are indicated.

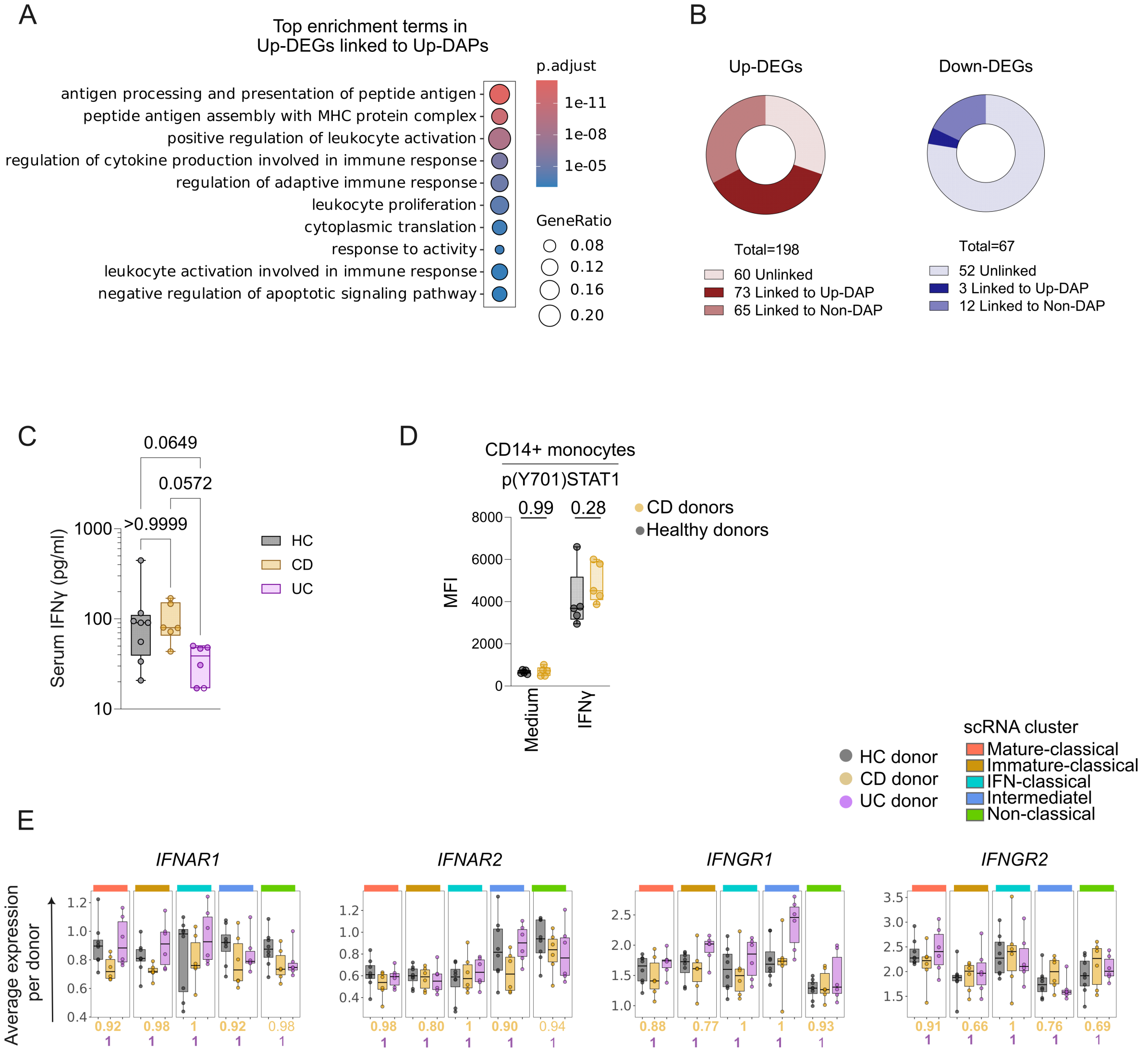

Supplemental Figure 6. (A) Top 10 significantly enriched Gene Ontology terms (Biological Process) for CD Up-DEGs that are linked to an Up-DAP. Gene ratio, representing the number of genes in the term divided by the total number of defining genes for that cluster, and adjusted *p*-value are indicated. **(B)** Numbers of CD DEGs that are either: linked to a non-DAP, linked to a DAP or unlinked to peaks. **(C)** Serum concentration of IFN-γ in donors from the blood monocyte scRNA-seq dataset. *p*-values denote the result of Kruskal-Wallis test with Dunn's correction **(D)** MFI of intra-nuclear STAT1(pY701) expression in $CD14^+$ monocytes (minus MFI of isotype control) from a separate cohort of treatment-naïve patients with active CD (n=5) and healthy controls (n=5) stimulated with either IFN-γ (100 U/mL), or medium alone for 15 minutes. *p*-values denote the result of two-way ANOVA with Šídák's correction. **(E)** Average expression of receptors for type I (*IFNAR1*, *IFNAR2*) and type II (*IFNGR1, IFNGR2*) interferons per donor per blood monocyte cluster. *p*-values denote the result of pseudobulk differential expression analysis performed in Figure 3. **(C), (D)** and **(E)** Box-and-whisker plots show the median (center line), 25th and 75th percentiles (box), and minimum and maximum values (whiskers). Individual data points are overlaid.

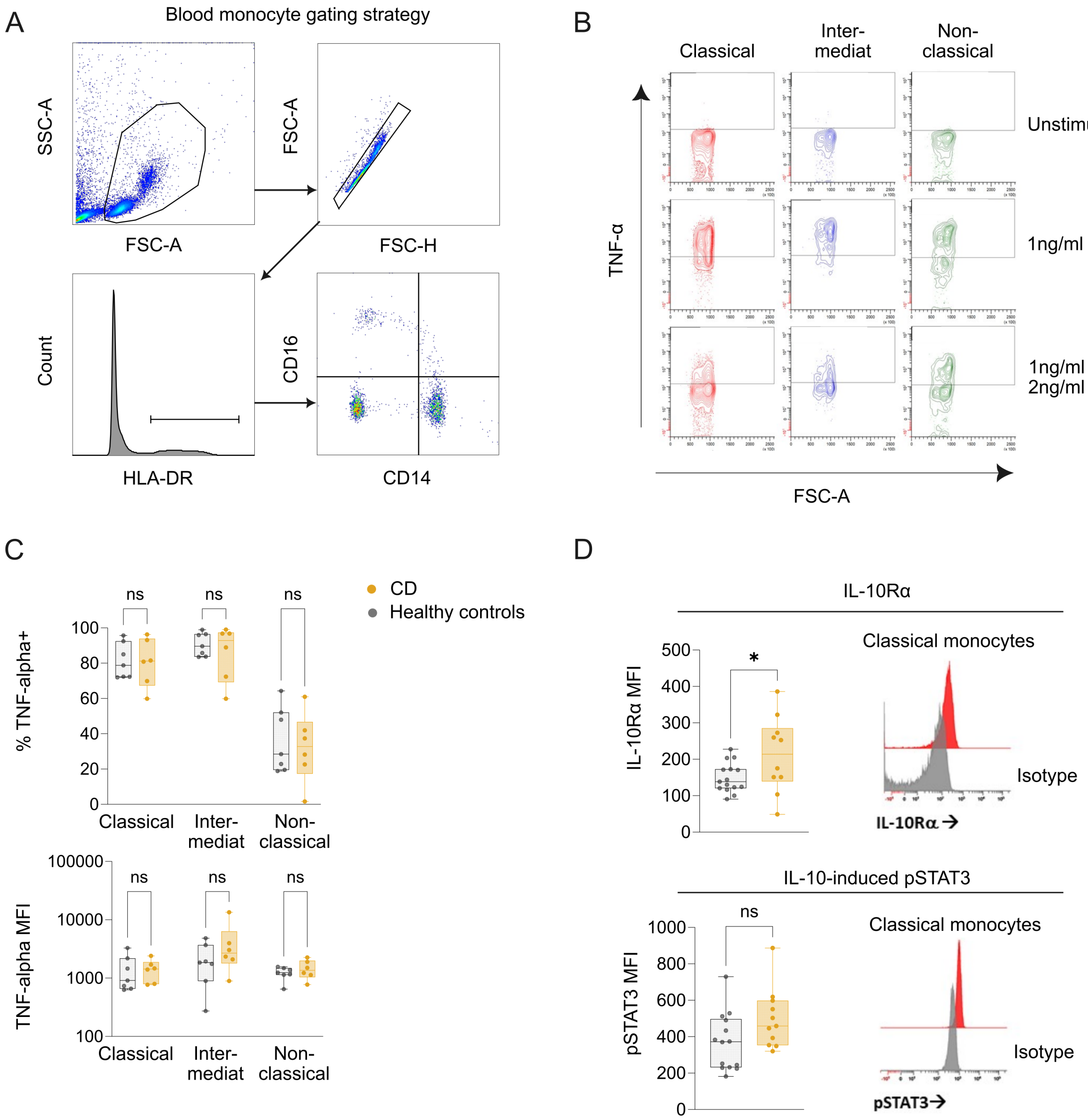

Supplemental Figure 7. (A) & **(B)** Flow cytometry gating strategy to identify LPS-induced intracellular TNF-α expression and its inhibition by 2 ng/mL IL-10 in classical (red), intermediate (blue) and non-classical (green) monocytes within PBMC cultures. **(C)** LPS-induced intracellular TNF-α expression in monocyte subsets from healthy controls (n=7) and patients with active Crohn's disease (n=6) in the absence of IL-10 expressed as both percentage positive cells and MFI (minus MFI of isotype control). Data were analysed statistically by two-way ANOVA with Šídák's correction. **(D)** MFI of IL-10Rα and IL-10-induced STAT3 (pY705) staining (minus MFI of isotype controls) in classical monocytes (identified as HLA-DR$^+$ CD14$^+$ CD16$^-$ in the gating strategy shown in **(A)**) from healthy subjects (IL-10Rα: n = 15; pSTAT3: n= 14) and patients with active CD (IL-10Rα: n = 10; pSTAT3: n= 11). Data were analysed statistically by an unpaired t-test. **(C)** and **(D)** Box-and-whisker plots show the median (center line), 25th and 75th percentiles (box), and minimum and maximum values (whiskers). Individual data points are overlaid. * = $p < 0.05$; ns = $p > 0.05$.

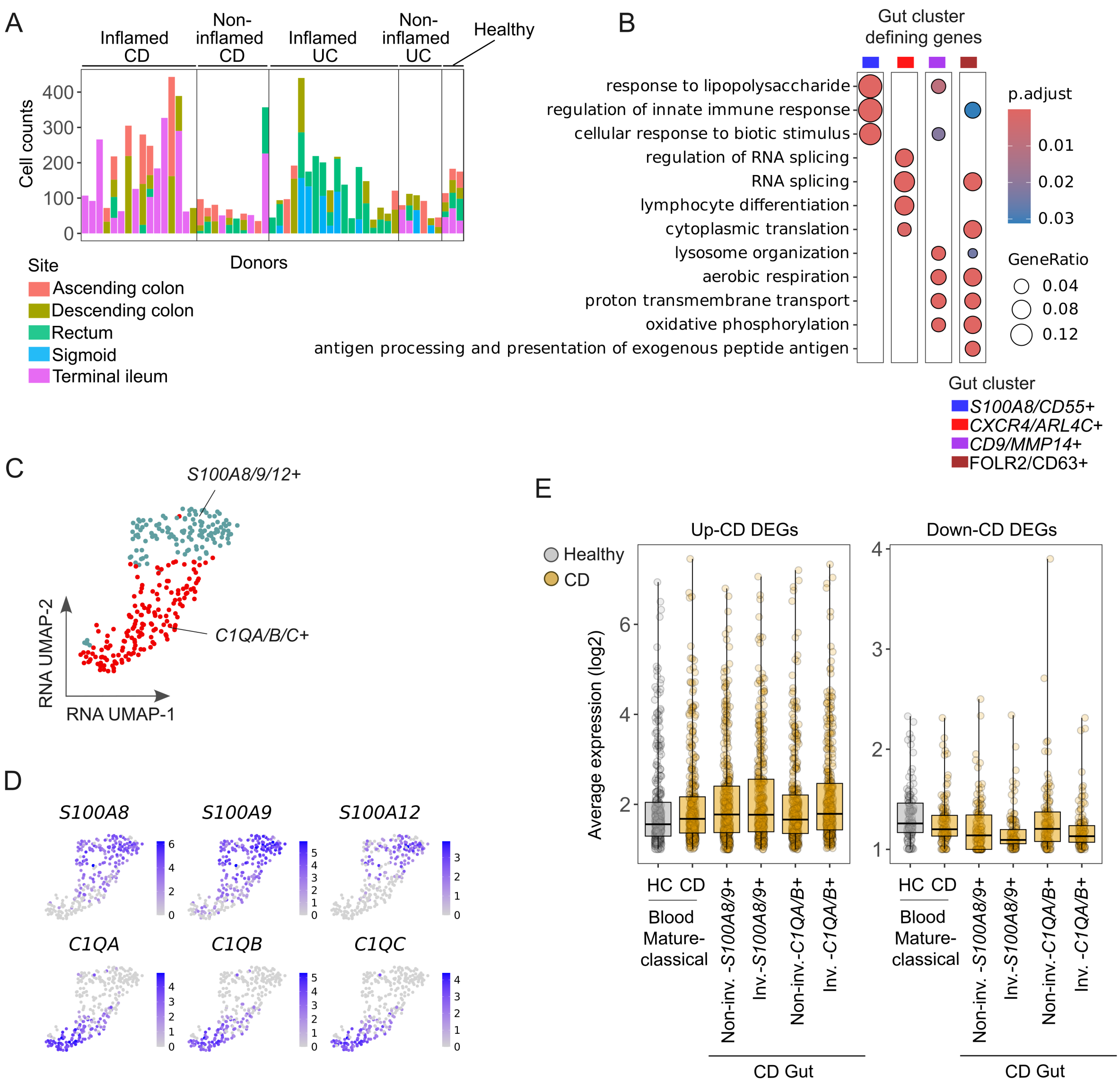

Supplemental Figure 8. (A) Cell counts per donor in the external intestinal monocyte/macrophage scRNA-seq dataset (2) derived from inflamed and non-inflamed biopsies of biologic-naïve patients with Crohn's disease (CD; n= 16) and ulcerative colitis (UC; n= 19), and healthy controls (HC; n=3). Contributions from different gut regions per donor are indicated. **(B)** Top 3 significantly enriched Gene Ontology (Biological Process) terms for gut cluster-defining markers after removal of redundant terms. Gene ratio, representing the number of genes in the term divided by the total number of defining genes for that cluster, and adjusted *p*-value are indicated. **(C)** UMAP projection of scRNA-seq data showing monocyte/macrophages from involved (n=5) and non-involved (n=4) regions of ileo-caecal surgical resections from five patients with CD. **(D)** Log-normalized expression of calprotectin (*S100A*) and *C1Q* genes. **(E)** Log_2 average cell expression of blood CD DEGs (identified in Figure 3) in blood mature classical monocytes from healthy controls and patients with CD, and in intestinal clusters from involved and non-involved regions of patients with CD. Box-and-whisker plots show the median (center line), 25th and 75th percentiles (box), and minimum and maximum values (whiskers). Individual data points are overlaid.

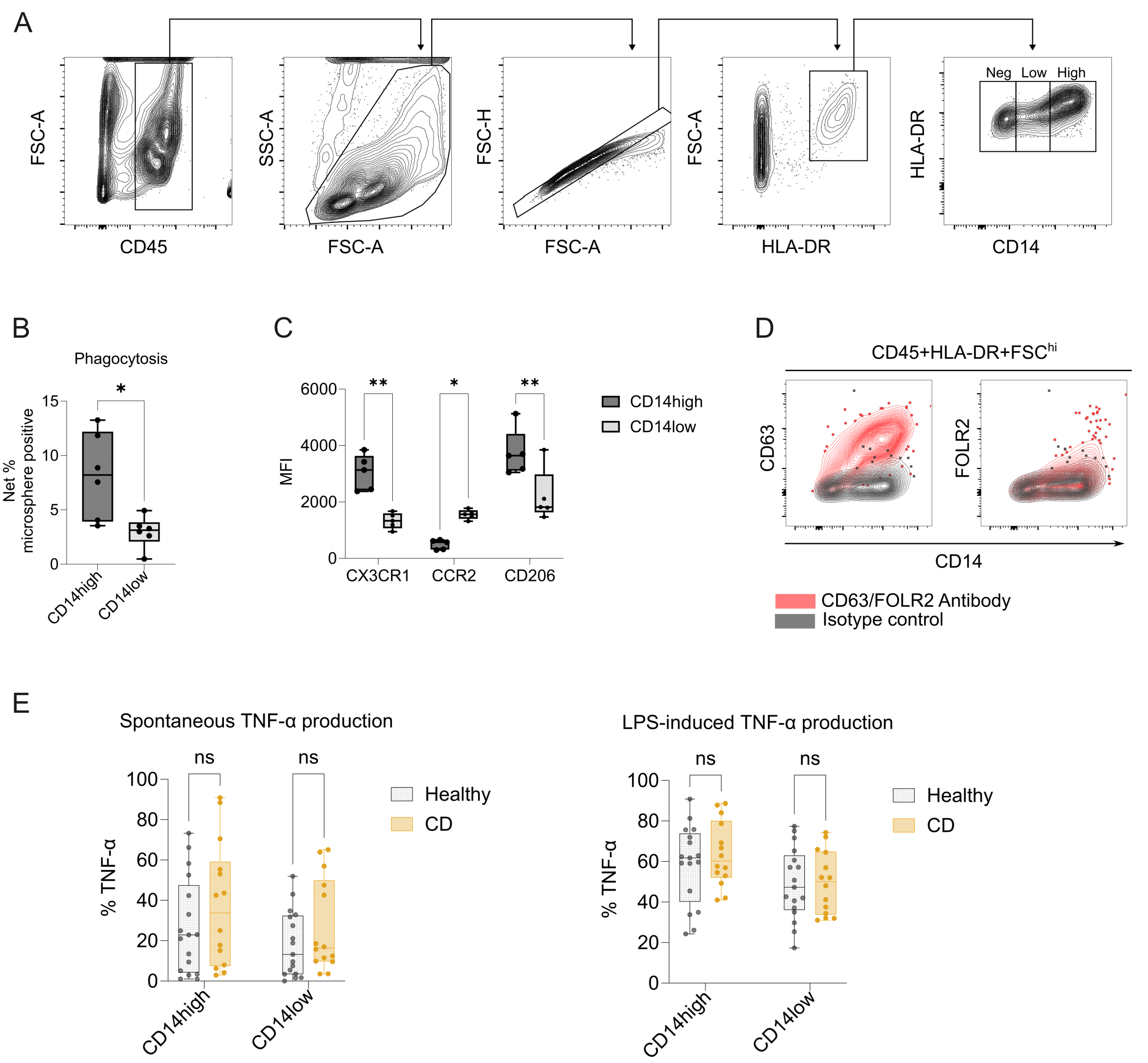

Supplemental Figure 9. **(A)** Flow cytometry gating strategy for the identification of CD14^{high}, CD14^{low} and CD14^{neg} cells within the CD45^{+}HLA-DR^{+}FSC^{int-hi} population of lamina propria mononuclear cells (LPMCs) enzymatically extracted from intestinal biopsies. **(B)** Phagocytic activity of CD14^{high}, and CD14^{low} cells. LPMCs were extracted from non-IBD intestinal biopsies (n=6) and cultured for 1.5 hours at 37°C or 4°C with fluorescent (FITC) microspheres and then analysed by flow cytometry. The proportion of microsphere-positive CD14^{high}, and CD14^{low} cells at 37°C (minus 4°C control) is shown. Data were analysed statistically by a paired t-test. **(C)** MFI of CX3CR1, CCR2 and CD206 (minus MFI of isotype control) in CD14^{high} and CD14^{low} cells from non-IBD samples (n=5). **(D)** Flow cytometry plots displaying CD63, FOLR2 and CD14 staining in CD45^{+}HLA-DR^{+}FSC^{int-hi} lamina propria cells from inflamed intestinal biopsies of a patient with active Crohn's disease. **(E)** 'Spontaneous' and LPS-induced intracellular TNF-α production in CD14^{high} and CD14^{low} cells from healthy subjects (n=17) or patients with active Crohn's disease (CD; n=14) quantified as the percentage of cells positive for TNF-α. **(C)** & **(E)** Data were analysed by two-way ANOVA with Šídák's correction. **(B)**, **(C)** & **(E)** Box-and-whisker plots show the median (center line), 25th and 75th percentiles (box), and minimum and maximum values (whiskers). Individual data points are overlaid. * = $p < 0.05$; ** = $p < 0.01$, ns = $p > 0.05$.

References

1. Humphreys DT, et al. Single cell sequencing data identify distinct B cell and fibroblast populations in stricturing Crohn's disease. J Cell Mol Med. 2024;28(9):e18344.
2. Thomas T, et al. A longitudinal single-cell atlas of anti-tumour necrosis factor treatment in inflammatory bowel disease. Nat Immunol. 2024;25(11):2152-65.
